# Persistent Organic Pollutants in Austrian Human Breast Milk Collected between 2013 and 2016

**DOI:** 10.3390/jox14010015

**Published:** 2024-02-07

**Authors:** Christina Hartmann, Andreas-Marius Kaiser, Wolfgang Moche, Stefan Weiss, Wolfgang Raffesberg, Sigrid Scharf, Klaudia Graf-Rohrmeister, Margarita Thanhaeuser, Nadja Haiden, Maria Uhl

**Affiliations:** 1 Environment Agency Austria, Spittelauer Laende 5, 1090 Vienna, Austria; andreas.kaiser@umweltbundesamt.at (A.-M.K.); wolfgang.moche@umweltbundesamt.at (W.M.); stefan.weiss@umweltbundesamt.at (S.W.); raffesberg@aon.at (W.R.); scharf.sigrid@outlook.de (S.S.); maria.uhl@umweltbundesamt.at (M.U.); 2 Ignaz Semmelweis Women’s Hospital, Bastiengasse 36-38, 1180 Vienna, Austria; klaudia.graf-rohrmeister@gesundheitsverbund.at; 3 Department of Pediatrics and Adolescent Medicine, Comprehensive Center for Pediatrics, Medical University of Vienna, Waehringer Guertel 18-20, 1090 Vienna, Austria; margarita.thanhaeuser@meduniwien.ac.at; 4 Department of Neonatology, Kepler University Hospital, Krankenhausstraße 26-30, 4020 Linz, Austria; nadja.haiden@kepleruniklinikum.at

**Keywords:** per- and polyfluoroalkyl substances, polybrominated diphenyl ethers, persistent organic pollutants, breast milk, daily intake

## Abstract

Breast milk holds an immense nutritional value as it contains health-promoting substances in a unique, optimal form. Additionally, breast milk’s significance extends to health and environmental protection, as it serves as an indicator of both maternal and infant exposure. In this study, breast milk samples collected in 2013 and in 2014–2016 from mothers in Vienna (Austria) were analysed for polybrominated diphenyl ethers (PBDE) and per- and polyfluoroalkyl substances (PFAS), as well as further substances which have been listed under the Stockholm Convention on Persistent Organic Pollutants (POPs) due to their persistent, bioaccumulative and toxic properties. The total concentration of the PBDE congeners in the samples (*n* = 18, sampled 2013) ranged from 0.055 to 52 ng/g lipid, and from 0.002 to 2.5 ng/g breast milk. In the pooled sample, the sum of PBDEs was detected at a level of 4.4 ng/g lipid. Based on the 2014–2016 study population, certain PFAS were detected in all samples (*n* = 40). Exposure to the sum of four specific PFAS including perfluorooctanesulphonate (PFOS), perfluorooctanoic acid (PFOA), perfluoro-n-nonanoic acid (PFNA) and perfluoro-1-hexanesulfonate (PFHxS) ranged between 0.014 and 0.12 ng/L breast milk. In the pooled sample, PFOS and PFOA were found in concentrations of 0.025 ng/g and of 0.045 ng/g, respectively. In addition, the first generation of POPs, mainly organochlorine compounds, was measured in a pooled sample of breast milk from participants sampled in 2014–2016 as part of the WHO/UNEP breast milk monitoring program and compared to the POPs measured in pooled samples collected in 1987/1988 and 1992/1993, respectively. Therefore, this paper demonstrates the effectiveness of the Stockholm Convention on POPs by comparing the Austrian results from the WHO/UNEP global breast milk study from 1987 to 2016. However, the data also show that, despite these reductions, health-relevant levels are still being reached, particularly in terms of children’s health when the presence of the new generation of POPs, such as PBDEs and PFAS, in human breast milk is taken into account.

## 1. Introduction

Breast milk is the ideal food for infants because it provides energy, nutrients and antibodies that can protect against many common childhood illnesses. The health benefits of breastfeeding are considerable: it reduces infant mortality and the risk of diseases such as *otitis media*, obesity, malocclusion and asthma. In addition, breastfeeding is also beneficial for women’s health [1,2]. The World Health Organisation (WHO) and United Nations Environment Programme (UNEP) therefore recommend that breastfeeding should be protected, promoted and supported when monitoring contaminants in human milk to assess the effectiveness of regulations [2].

Persistent organic pollutants (POPs) are organic substances (mostly) of anthropogenic origin that have persisted in the environment for many decades. They can be widely distributed throughout the globe via air, water or migratory species. Furthermore, they accumulate in living organisms and pose a risk to human health and ecosystems [3,4]. Infants are particularly vulnerable since early-life exposure to harmful substances can be linked to adverse effects on later development [5]. However, due to the lipophilic properties of many xenobiotics and POPs, they are both present in human breast milk, resulting in infant exposure [6,7].

Polybrominated diphenyl ethers (PBDEs) are a group of brominated congeners, which have been utilised as flame retardants for several decades. They were produced in high volumes and used in a wide range of consumer products such as TVs, computers, mobile phones, electrical equipment and textiles, as well as in construction materials, cars and plastic products [8]. Generally, PBDEs were manufactured commercially at three different bromination levels including pentaBDE, octaBDE and decaBDE as technical mixtures of which decaBDE made up the majority of the global PBDE market [9]. As PBDEs are not chemically bound to the polymer structure of the products, migration into the environment is possible [10]. Thus, PBDEs can be found in different environmental media [11,12,13,14], as well as human matrices such as blood, cord blood and breast milk [8,13,15,16,17,18]. PBDEs can affect the liver, thyroid hormone homeostasis and the reproductive and nervous system. In addition, epidemiological studies supported an association between exposure to PBDEs and hyperthyroidism and neuropsychological function [19,20,21]. Several PBDEs were identified to be persistent, bioaccumulative and toxic, and are thus be classified as POPs. Due to their properties, hexaBDE and heptaBDE (the main components of commercial octaBDE); tetraBDE and pentaBDE (the main components of commercial pentaBDE); as well as decaBDE (commercial mixture) are listed under Annex A of the Stockholm Convention and are therefore banned with specific restrictions [22].

Per- and polyfluoroalkyl substances (PFAS) are a group of synthetic chemicals consisting of several thousand substances, of which the two perfluoroalkyl acid (PFAA) sub-groups perfluorocarboxylic acids (PFCAs) and perfluoroalkane sulfonic acids (PFSAs) are currently the best studied [23,24,25]. The extremely persistent PFAAs consist of a fully fluorinated alkyl chain with 4–18 carbon atoms with either a carboxylic or sulfonic terminal functional group [26]. Due to their unique physical-chemical properties such as being both oleophobic and hydrophobic [27], PFAS are used in many processes and products including repellents for leather, textiles and papers, inks, firefighting foams and hydraulic oils [28], but are also used in food packaging materials, kitchenware, insecticides and cosmetics [29]. PFASs can be released during the manufacturing process, with their use and application being ubiquitous, and are globally found in the environment, but also in wildlife and humans [23,26]. Two PFASs, perfluorooctanoic acid (PFOA) and perfluorohexane sulfonic acid (PFHxS), as well as their salts and related compounds are included in the list of restricted chemicals of the Stockholm Convention of POPs in Annex A and perfluorooctane sulfonic acid (PFOS), and its salts and perfluorooctane sulfonyl fluoride (PFOSF) are included in Annex B [22]. Additionally, long-chain PFCAs, their salts and related compounds are currently proposed for listing under the Stockholm Convention [30]. The main exposure source is food, especially fish, seafood, milk products, fruit, meat and eggs [23,31]. In humans, the half-lives of specific PFASs are several years, amounting to approximately 3.8 years for PFOA, 5.4 years for PFOS and 8.5 years for PFHxS [32]. Research was primarily conducted in blood [23,27,28,33,34], but also in urine [35,36,37] and in breast milk [38,39,40,41]. Extensive toxicity data are primarily available for PFOS and PFOA, showing the effects on the liver, the immune system, the developmental system and the hormone system, as well as a carcinogenic potency [5,23,42]. In embryos, PFAS exposure occurs via the placenta and mostly via breast milk in newborns [23].

The European Food Safety Authority (EFSA) CONTAM Panel lowered the tolerable weekly intakes (TWI) for PFOSs and PFOAs tremendously from 2008 to 2020, currently suggesting a TWI of 4.4 ng/kg body weight (bw)/week (w) for the sum of PFOA, perfluorononanoic acid (PFNA), PFHxS and PFOS [23,27,43].

The aim of this study was to investigate the exposure to POPs and other xenobiotics in the breast milk samples of Austrian mothers collected in 2013 and in 2014–2016. Further, the results of the Austrian data of the WHO/UNEP milk survey from 1987/88 and 1992/93 were compared with the new data. In addition, considerations were made with regard to a possible risk in terms of children’s health.

## 2. Materials and Methods

This study in Austrian human breast milk comprises three parts: (i) a pilot study to investigate 20 PBDE congeners in 18 samples and 14 PFAS in 21 samples collected in 2013, (ii) a follow-up study to investigate 25 PFAS in 40 samples collected in 2014–2016 and (iii) the analysis of different POPs in a pooled breast milk sample comprising 34 individual samples from the study population collected in 2014–2016 in the framework of the WHO/UNEP breast milk monitoring programme (see Table 1 for more details).

### 2.1. Samples and Study Populations

#### 2.1.1. Pilot Study

In the pilot study, 21 breast milk samples were collected in 2013 from Austrian mothers at the Semmelweis Woman’s Hospital in Vienna, Austria. The samples were given 11–81 days (mean: 28) after delivery. Data on education, nutrition, employment before pregnancy, residence and use of water-proof textiles and non-stick cookware were collected via questionnaires. The study population included 21 women aged 22–40 years (mean: 30). The deliveries occurred between weeks 37 and 42, with the majority of mothers delivering at weeks 38–39. While PFASs were analysed in all 21 samples, PBDE congeners were analysed in only 18 breast milk samples due to the partially limited sample volume available. The pilot study was approved by the ethics commission of the City of Vienna (number: EK-12-244-VK). Details on the study population are provided in Table 1.

#### 2.1.2. Follow-Up Study

As a follow-up, breast milk samples were collected from 40 mothers aged 21–36 years (mean: 29) between 2014 and 2016 in Vienna, Austria, in collaboration with the Department of Pediatrics and Adolescent Medicine, Comprehensive Center for Pediatrics, Medical University of Vienna, Austria. The samples were provided 21–56 days (mean: 32) after delivery. Details are provided in Table 1. Similar to the pilot study, information on the mothers was collected via questionnaires. A total of 25 PFASs were analysed in the samples. The follow-up study was approved by the ethics commission of the Medical University of Vienna (number: 1370/2014).

#### 2.1.3. WHO/UNEP Breast Milk Monitoring Programme

As part of the WHO/UNEP breast milk monitoring programme for the evaluation of the efficiency of the Stockholm Convention [44], one pooled breast milk sample containing individual samples from 34 mothers aged 21–36 years (mean: 28 years) from the follow-up study population (see above) sampled between 2014 and 2016 in Vienna, Austria, was analysed for various POPs. The individual breast milk samples were provided 3–8 weeks (mean: 4.6 weeks) after delivery. WHO standardised questionnaires were used to collect data on diet, employment status and residence status, among others.

### 2.2. Chemical Analysis

#### 2.2.1. Analysis of PBDE Congeners (Pilot Study)

In the pilot study, the concentrations of 20 PBDE congeners (see Table S1 in the Supplementary Information) in breast milk were analysed via gas chromatography coupled with high-resolution mass spectrometry (GC-HRMS), in accordance with method 1614 published by the U.S. Environmental Protection Agency [45] and using Soxhlet extraction followed by a three-step liquid column chromatographic clean-up. For the sample preparation, 10 g breast milk was spiked with a mix of 12 ^13^C-labelled surrogate standards (Wellington Laboratories, USA) (see Table S2 in the Supplementary Information) followed by freeze drying and Soxhlet extraction with toluene. The volume of the extract was reduced to less than 50 mL using a rotary evaporator, transferred to a 50 mL volumetric flask and topped up to 50 mL with toluene. In total, 2 mL of the extract was used for the determination of lipid content in the sample and the remaining 48 mL was evaporated to approx. 5 mL. The following clean-up was carried out using a semi-automatic liquid column clean-up system. The extract was applied to a column (3 cm × 20 cm) filled with celite and sulfuric acid (1:1) and eluted directly with n-hexane onto a column (1 cm × 20 cm) filled with silica/44% sulfuric acid, silica and silica/33% 1N NaOH. In a further step, a second elution was carried out with n-hexane directly in the next column filled with Alox. The PBDE fraction was eluted with n-hexane and dichloromethane (7:3) and evaporated in a Turbovap to approx. 4 mL. After the transfer to a conical vial, the solution was evaporated under a nitrogen stream to a volume of 100 µL. A ^13^C-labelled injection standard (^13^C-BDE-118) was added, and the solution was transferred into an autosampler vial with a conical insert.

The measurement was carried out with a gas chromatograph coupled to a MAT 95 XP high-resolution mass spectrometer (Thermo Fisher Scientific, Bremen, Germany). For the separation of Tri- to HeptaBDE a DB5MS (J&W) 60 m, 0.25 mm and 0.25 µm was used; for the separation of Octa- to DecaBDE, a Rtx 5 (Phenomenex, Torrance, CA, USA) 15 m, 0.25 mm and 0.25 µm was used. The injector for both columns was a PTV in solvent vent mode. The mass spectrometer was used in MID mode with a mass resolution of more than 8000. The quantification was performed in accordance with an isotope dilution method with TargetQuan 2.0 software. Recovery rates, which were used for the recovery correction of the results, were calculated for each individual sample using ^13^C-labelled surrogate standards (the assignment of the native standards to the labelled standards is provided in Table S3 of the Supplementary Information). The respective limits of detection (LODs) were calculated based on validation data corrected by sample intake and recovery rate. The limits of quantification (LOQs) were calculated as mean blank plus the threefold standard deviation. The respective LODs and LOQs of the analysed PBDE congeners are listed in Table S1 in the Supplementary Information. As national reference laboratory for halogenated POPs in feed and food, the Environment Agency Austria is obliged to participate in proficiency tests twice a year.

#### 2.2.2. Analysis of PFASs (Pilot Study)

The analysis of the concentrations of 14 PFASs (see Table S1) in the breast milk samples collected in 2013 was conducted via high-performance liquid chromatography–tandem mass spectrometry (HPLC-MS/MS) for simultaneous determination. The accredited analytical method was based on a method published by Kuklenyik et al. [46] and was adapted to an off-line method including an additional dispersive clean-up with charcoal. All reagents were cleaned via solid-phase extraction (SPE) with Oasis hydrophilic/lipophilic-balanced (HLB) cartridges. For sample preparation, 10 g breast milk was weighed into polypropylene tubes (Sarstedt, Nümbrecht, Germany) and spiked with an isotopically labelled surrogate mixture including ^13^C-labelled standards for PFBA, PFHxA, PFOA, PFNA, PFDA, PFDoDA and PFOS (Wellington Laboratories Inc., Guelph, ON, Canada). After addition of 40 mL 0.1 M formic acid (HFA) (Merck, Darmstadt, Germany), samples were treated in an ultrasonic bath for 20 min. For the preparation of the blanks, 10 g of cleaned tap water was used. The samples were applied to cartridges (Oasis HLB 500 mg, Waters Corporation, Milford, MA, USA) which were treated with methanol (MeOH) (Pestinorm, VWR International, Leuven, Belgium) and HFA. The cartridges were washed with 2 × 6 mL HFA, followed by 6 mL HFA/MeOH (6:4) and aqueous ammonia solution (NH_4_OH)/HPLC water (1:100) (Merck, Darmstadt, Germany), and were dried for 30 min using nitrogen. After elution with 10 mL NH_4_OH/acetonitrile (ACN) (1:100) (Optigrade, Promochem, LGC Standards, Wesel, Germany) and restriction to a volume of 200 µL, using nitrogen, 50 µL acetic acid (HAc) (Merck, Darmstadt, Germany) was added and the samples was filled up with ACN volume of 500 µL. After centrifugation for 10 min at 4,000 rpm, the samples were transferred to centrifuge tubes with 25 mg Supelclean ENVI-Carb 120/400 (Sigma-Aldrich Co. LLC., St. Louis, MO, USA) and centrifuged again at 10,000 rpm for 20 min. Afterwards, 350 µL of the supernatant was put into polypropylene tubes, and 350 µL HPLC water (Optigrade, Promochem, LGC Standards, Wesel, Germany) and an internal standard solution were added. Finally, samples were transferred to polypropylene autosampler vials.

PFAS levels in the breast milk samples were measured using an Agilent Technologies 1290 Infinity Series (Agilent Technologies, Santa Clara, CA, USA) as a HPLC system and an AB Applied Biosystem MDS SCIEX 4000 QTRAP LC/MS/MS System (AB Sciex Technologies, Framingham, MA, USA) as an MS detector system. The detection was performed through specific mass transitions in electrospray ionisation (ESI)-negative mode and the quantification in multiple reaction monitoring (MRM) mode. The analytical column used was a Luna 5 µm C18(2) 100 mm × 2 mm (Phenomenex, Torrance, CA, USA). The separation of measured PFASs was conducted with a 25 min gradient elution method using 10 mMol ammonium acetate in water and MeOH.

The external calibration included 14 concentration levels ranging between 0.1 and 20 ng/mL. The samples were measured twice with injection volumes of 10 µL and 2 µL, respectively. The analytical results are presented as their means corrected by blanks and recoveries of the corresponding surrogate standards. Recovery rates in the extracts can be found in Table S2 of the Supplementary Information. LOQs were determined according to DIN 32645:2008-11 [47] and are listed in Table S1.

#### 2.2.3. Analysis of PFASs (Follow-Up Study)

In 2021, breast milk samples collected in 2014–2016 were measured using an adapted HPLC-MS/MS including a total of 25 different PFAS (see Table S1). After addition of an isotope-labelled surrogate standard mixture, 5 mL of acetonitrile and 150 µL of 0.5 M formic acid the samples were extracted via ultrasonic extraction. In addition to the surrogate standards used in the pilot study, further ^13^C-labelled standards for PFPeA, PFUnDA, PFTeDA, 6:2-FTS and GenX as well as a ^18^O-labelled PFHxS standard were used, which were also provided by Wellington Laboratories (Guelph, ON, Canada). Purification of the extract was performed by adding 2 mL of n-hexane and 100 mg of EnviCarb powder. After each purification step, samples were vortexed for 1 min and centrifuged for 15 min at 4000 rpm. After constriction of the solvent to 250 µL, extracts were filled up to 500 µL with methanol/water 80/20 *v/v* and were measured using HPLC-MS/MS. Extracts were measured on a Waters LC-MS/MS system comprised of an Acquity I-Class UPLC and a Xevo TQ-S mass spectrometer. Chromatographic separation was performed on 100 mm × 2.1 mm Acquity UPLC BEH C18 column (Waters, Milford, MA, USA), and an additional Waters Isolator Column was used to reduce the instrumental blank values. A gradient elution was performed using Milli-Q water/methanol 98/2 *v/v* modified with 2 mM ammonium acetate and methanol as eluents. External calibration levels ranged between 0.01 and 10 ng/mL. A sample volume of 2 µL was injected into the system. As in the pilot study, procedural blanks were subtracted from each batch, and the found concentrations were corrected with the recoveries of the corresponding isotopically labelled standard. LODs and LOQs are presented in Table S1, and the recovery rates in the extract are provided in Table S2 of the Supplementary Information.

#### 2.2.4. Analyses of POPs

In the framework of the WHO/UNEP monitoring programme, the Austrian pooled breast milk sample was analysed for different POPs in a WHO reference laboratory. After sampling, the 34 individual breast milk samples were pooled at the laboratory of the Environment Agency Austria according to the guidance of the WHO [44] and were shipped in glass bottles to the respective reference laboratory in Germany which conducted the analyses, except for PFASs, which were analysed in a Swedish laboratory [48]. An overview on the substances analysed including the LODs and LOQs is provided in Table S1.

### 2.3. Statistical Analysis

Statistical analysis was conducted using IBM^®^ SPSS^®^ Statistics Version 21 and R Version 4.2.0. Descriptive statistics (ranges, means, medians, 95th percentiles (P95)) were determined for PBDE congener concentrations in ng/g lipid and ng/g breast milk, for PFAS concentrations in µg/l breast milk, for daily PBDE intakes in µg/kg body weight (bw)/d and for daily PFAS intakes in ng/kg bw/d. For data treatment, measured concentrations below LOQs were set to LOQs/2 and concentrations below LODs were set to 0. Correlations between different parameters such as breast milk exposure, age or body weight were analysed using Spearman’s rank correlation.

### 2.4. Estimation of Daily Intakes in Infants

To assess the exposure of infants to PBDE congeners and PFASs via breast milk, the average daily intake and the high daily intake were calculated based on the measured PBDE and PFAS concentrations. Similarly, the average and high daily intake were estimated based on the results of the analyses of the WHO’s pooled breast milk sample.

Based on EFSA (2011) [21], a mean daily breast milk consumption of 800 mL and a high daily breast milk consumption of 1200 mL for an infant with an average body weight of 6.1 kg were used to estimate exposure. The use of these assumptions allows to complete a comparison with the results of other international exposure assessments. In addition, where not available, we calculated the median and mean daily intakes based on PFOA and PFOS breast milk levels reported in studies conducted in European populations to also allow for comparisons to be made.

## 3. Results and Discussion

### 3.1. PBDE Congener Levels

#### 3.1.1. Exposure

The results of the exposure to the PBDE congeners in the pilot study (2013) expressed in ng/g lipid and ng/g breast milk as well as the exposure to the PBDE congeners in the Austrian WHO/UNEP’s pooled sample (2014–2016) expressed in ng/g lipid are presented in Table 2.

**Figure 1:**
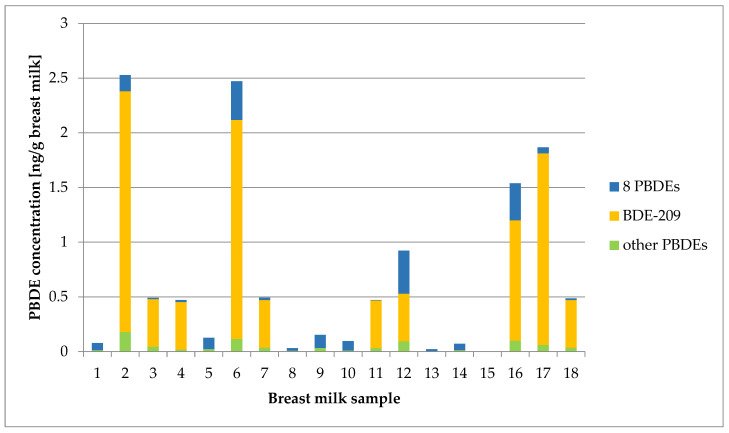
Distribution levels of BDE-209, the sum of eight PBDEs and other PBDEs in breast milk samples of 18 Austrian mothers. Eight PBDEs: Σ of BDE-28, BDE-47, BDE-66, BDE-85, BDE-99, BDE-100, BDE-153 and BDE-154. Other PBDEs: Σ of BDE-49, BDE-77, BDE-118, BDE-126, BDE-139, BDE-181, BDE-183, BDE-196, BDE-197, BDE-203 and BDE-207.

All 18 mothers studied in the pilot study showed detectable concentrations of PBDE congeners in their breast milk samples with varying exposure patterns and levels. Of the 20 PBDEs measured, only two congeners were not detected in any of the samples: 2,2′,3,4,4′-pentabromodiphenyl ether (BDE-85) and 2,2′,3,4,4′,5,6-heptabromodiphenyl ether (BDE-181). The highest concentration was found for 2,2′,3,3′,4,4′,5,5′,6,6′-decabromodiphenyl ether (BDE-209) with 43 ng/g lipid (2.2 ng/g breast milk) in one sample. The total concentration of the PBDE congeners (sum PBDE) in the samples ranged from 0.055 to 52 ng/g lipid (median: 11 ng/g lipid), and from 0.002 to 2.5 ng/g breast milk (median: 0.47 ng/g breast milk). The results are shown in Figure 1. The main contributor to the total PBDE congener exposure in the majority of the breast milk samples was BDE-209, a decabromodiphenyl ether (decaBDE). It was detected in 55.6% of the 18 breast milk samples and contributed to the total exposure in fractions ranging from 47.4 to 93.8%.

For the different PBDE congeners, statistically significant correlations were identified. They are shown in Table S4 in the Supplementary Information. Most of triBDE, tetraBDEs, pentaBDEs and hexaBDEs were significantly correlated, whereas higher congeners were less likely to be correlated. Vice versa, the majority of the PBDE congeners of the groups of heptaBDEs, octaBDEs, nonaBDE and decaBDE showed significant correlations.

In the WHO/UNEP’s pooled sample, almost all PBDEs measured were detected at levels above the LOQ, except 2,3′,4,4′-tetrabromodiphenyl ether (BDE-66), 2,4,4′,6-tetrabromodiphenyl ether (BDE-75), 3,3′,4,4′-tetrabromodiphenyl ether (BDE-77) and 2,3,3′,4,4′,5,6-heptabromodiphenyl ether (BDE-190), which could not be detected. The sum concentration of the PBDE congeners in the sample was 4.38 ng/g lipid.

#### 3.1.2. European Studies on PBDEs in Human Breast Milk

Eight PBDE congeners, including 2,4,4′-tribromodiphenyl ether (BDE-28), 2,2′,4-tribromodiphenyl ether (BDE-47), BDE-66, BDE-85, 2,2′,4,4′,5-pentabromodiphenyl ether (BDE-99), 2,2′,4,4′,6-pentrabromodiphenyl ether (BDE-100), 2,2′,4,4′,5,5′-hexabromodiphenyl ether (BDE-153) and 2,2′4,4′5,6′-hexabromodiphenyl ether (BDE-154) were analysed the most in previous European and international studies. In this pilot study, exposures to these substances were n.d.–12.5 ng/g lipid (median: 1.06 ng/g lipid) and n.d.–0.39 ng/g breast milk (median: 0.056 ng/g breast milk), respectively.

As shown in Table S5, various European studies have investigated exposure to at least eight PBDE congeners as well as BDE-209 in breast milk between 1996 and 2016. The median BDE-28 concentration of 0.048 ng/g lipid assessed in this pilot study (2013) did not differ much from the values in other European countries in the years before or after. Merely in Finland in 1997–2001 [49], Norway in 2000–2001 [50] and Germany in 2005 [51], the BDE-28 concentrations were slightly higher in the past. A similar pattern was observed in Sweden [52], Finland [49], Norway [50], Poland [53], France [54] and the United Kingdom [55,56] for BDE-47, BDE-99 and BDE-153, where the levels in breast milk were comparably slightly higher to more than twice as high in the past. Except for France and Poland, this pattern was also similar for BDE-100. However, the median BDE-154 levels in Austria were higher in this study compared to all other European countries, except for the United Kingdom in 2010 [55]. Moreover, median BDE-209 (9.5 ng/g lipid) levels in breast milk were up to 4 times higher in Austria compared to other European countries. Concerning the comparability of the data, Lee et al. [57] reported that no statistically significant changes in PBDE concentrations were observed between lactation < 7 days, 15 days or 30 days after delivery. Daniels et al. [58] reported that neither the ΣPBDE concentration nor the lipid content in the milk changed statistically significantly between the 3rd and 12th month postpartum, i.e., no differences between PBDE levels were overserved from month to month.

The 3rd Global Monitoring Report (GMR) showed decreasing trends for some PBDEs in Europe, e.g., for BDE-47 [59]. BDE-209 (decaBDE), which was used as a replacement for penta- and octa-BDE mixtures that were regulated earlier and showed a declining trend in Europe, showed an increasing trend or no changes in concentrations [59].

#### 3.1.3. Associations with Individual Exposure Data and Health Parameters

Higher BDE-49, BDE-66, BDE-139 and BDE-153 levels were statistically significantly correlated with lower birth weights (rs = −0.55, *p* = 0.018; rs = −0.56, *p* = 0.015; rs = −0.56, *p* = 0.015 and rs = −0.48, *p* = 0.046, respectively). However, due to the small sample size, these results should be interpreted with caution, and further investigations are needed to strengthen these results. Some other studies, on the other hand, support these findings [60,61,62,63].

Further statistically significant associations/differences between PBDE breast milk levels and any of the individual exposure data (e.g., egg or fish consumption) were not identified.

#### 3.1.4. Risk Assessment for PBDEs

The average and high daily intake for the sum of PBDE congeners analysed in the breast milk samples in this pilot study were calculated based on EFSA (2011) [21], assuming a breast milk consumption of 800 mL (average intake) and 1200 mL (high intake) per day for infants below 6 months (Table 2). The medians of the average daily intakes of BDE-28, -47, -99, -154 and -183 were within the range reported in other studies [21]. For BDE-209, both the median as well as the highest calculated daily intake in this study were higher than the range reported in previous studies [21]. It is worth noting that some studies indicate adverse cognitive outcomes among children, which are associated with early life exposure to penta-BDE mixtures, and provide new evidence for the potential neurotoxicity of BDE-209 [64,65,66]. The calculated median average daily intakes for BDE-100 and -153 were within the range of previous studies. As shown in Table 2, the same pattern was observed for high daily intakes. The data show that the average and high daily intakes in the Austrian study population in 2013 were mostly comparable to those in other European countries (median within the range), but some individuals had even higher daily intakes above the ranges indicated.

### 3.2. PFAS Levels

#### 3.2.1. Exposure

In the pilot study completed in 2013, of the 14 PFAS investigated, only PFOS and PFOA were detected at levels above the LOQ (see Table 3). PFOS was found in 100% and PFOA in 52% of the samples.

In the follow-up study during 2014–2016, 19 of the 25 PFAS analysed were detected in at least one of the 40 human breast milk samples (see Table 3). PFOA, PFHxS, PFOS and PFNA were the PFASs with the highest detection frequencies (85–100%), followed by perfluorodecanoic acid (PFDA, 45%) and perfluorohexanoic acid (PFHxA, 33%). Perfluoropentane acid (PFPeA), perfluoroheptane sulfonate (PFHpS), perfluorononane sulfonate (PFNS), N-ethyl-perfluorooctane acidic acid (EtFOSAA) and the two fluorotelomer sulfonates (FTS)—4:2-FTS and 8:2-FTS—were not detected in any sample.

The LODs and LOQs of the PFASs determined in the follow-up study of 2014–2016 are notably lower compared to those of the pilot study of 2013 (cf. Table S1 in the Supplementary Information), which is due to the application of an improved analytical method. This clearly shows why considerably higher detection rates were observed in the follow-up study.

In the pooled breast milk sample investigated in the frame of the WHO/UNEP monitoring programme, two of the eight PFASs investigated were detected to consist of PFOSs and PFOAs at concentrations of 0.025 ng/g and of 0.045 ng/g breast milk, respectively.

#### 3.2.2. European Studies on PFASs in Human Breast Milk

As shown in Table S6, PFOS concentrations in the breast milk of Austrian mothers sampled in 2013 were quite comparable to those of other European countries, especially from Sweden (2004) [48], Germany (2006) [67], France (2007) [38], Spain (2007–2008) [68] as well as Belgium (2009–2010) [6], mostly with detection frequencies of or near to 100%. The median PFOS levels in breast milk of these previous studies ranged from 0.080 to 0.17 µg/L compared to our study with a median of 0.11 µg/L. Guerranti et al. [69] reported the highest PFOS concentration detected in a breast milk sample in Europe with 4.43 µg/L in Siena (Italy). However, in this follow-up study, the median PFOS level was 0.012 µg/L lower, which is probably related to the PFOS restrictions showing their effectiveness—as described in the 3rd Global Monitoring Report [59].

In this study, the PFOA exposure levels in breast milk samples were mostly lower compared to other European studies (see Table S6).

#### 3.2.3. Risk Assessment for PFAS

In 2018, the EFSA suggested a tolerable weekly intake (TWI) of 6 ng/kg bw/week for PFOA and of 13 ng/kg bw/week for PFOS [43]. In 2020, EFSA revised their scientific opinion and proposed a TWI of 4.4 ng/kg bw/week intake for the sum of the following four PFASs: PFOA, PFNA, PFHxS and PFOS. This TWI is based on the BMDL_10_ for 1-year old children and should prevent reaching a stage of physical burden in mothers resulting in concentrations in breast milk that are associated with negative health effects (such as a decrease in vaccine response). Since the higher exposure in breastfed children was considered in the TWI deviation, the TWI should not be used for comparisons with the intake of infants [23]. However, to obtain the first estimation of the calculated daily intake of infants based on the measured results in breast milk, these weekly intakes were discussed based on the TWI.

For the pilot study of 2013 and the follow-up study of 2014–2016, the (2011) [21] calculated weekly intake for infants in this study population according to EFSA is presented in Table 3.

The comparison of the weekly intake calculated with the proposed EFSA TWI showed that the average intake was exceeded 14- to 65-fold and was even 22 to 97 times higher based on high intake.

In this follow-up study, newer PFASs as well as substitutes of legacy PFAS (e.g., hexafluoropropylene oxide dimer acid (GenX), dodecalfluoro-3H-4,8-dioxanonanoate (DONA) and 6:2 chlorinated polyfluorinated ether sulfonate (Cl-PFESA)) were investigated in Austrian breast milk and were, to the best of our knowledge, also detected for the first time (Table 3). This highlights that they, as well as other PFASs, must also be considered in the exposure and risk assessment since they have already arrived in the human body.

**Figure 2:**
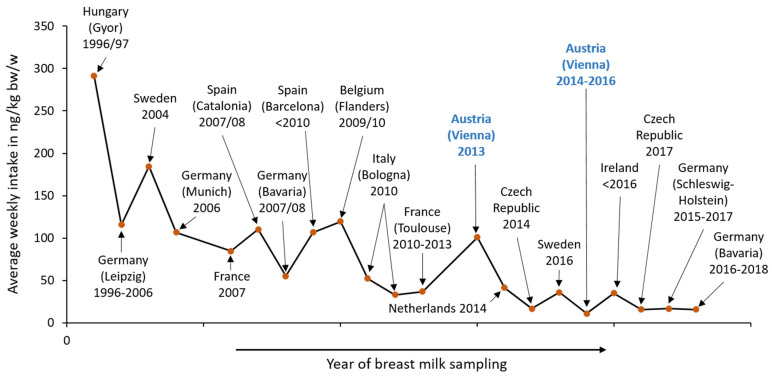
Average weekly intake of PFOSs in ng/kg bw/w in Europe from 1996 to 2018 (mean values, except for Austria (2013), The Netherlands (2014) and Ireland (<2016), for which medians are shown). In blue: results of the present study.

Based on the available median/mean PFOS and PFOA levels in breast milk reported in several European studies, weekly intakes for all studies as well as the average weekly intake for the years before 2009, between 2009 and 2013, and from 2014 to 2018 were calculated. The results are presented in Table S6 in the Supplementary Information. The data show that the restriction of PFOSs caused a decreasing trend for this chemical (see Figure 2). However, the current exposure data demonstrate that PFAS levels in human breast milk are still too high; therefore, future monitoring studies are necessary to verify if this downward trend is truly continuous, and they are also needed to collect data for the recognition of new threats and to avoid exposure to toxic substitutes.

### 3.3. POPs Levels

#### 3.3.1. Exposure

In the frame of the WHO/UNEP breast milk monitoring programme, various POPs were detected in the breast milk pool sample collected between 2014 and 2016: dioxins and furans (PCDD/Fs); dioxin-like polyfluorinated bisphenyls (PCBs) and indicator PCBs; chlordane; dieldrin; substances of the dichlorodiphenyltrichloroethane (DDT) group; substances of the heptachlor group; hexachlorobenzene (HCB); β-hexachlorocyclohexane (β-HCH); α-hexabromocyclododecane (α-HBCD); and chlorinated paraffins. Results are provided in Table S7. In general, the measured levels of POPs in the pooled breast milk sample were inconspicuous; thus, they correspond to the ubiquitous background contamination of these substances.

The concentrations of chlordane, heptachlor, HCB, α-HBCD and the sum of the compounds of the DDT group were within the range of the WHO study’s concentrations (2007) [70] and/or also within the range of other studies carried out in Europe and worldwide [71,72]. The DDT level detected was below the German reference value of 500 ng/g lipid [73] and far below the biomonitoring equivalent (BE) value of 2300 ng/g lipid [74]. Further, HCB was far below the German reference value of 60 ng/g lipid [73]. For total HBCD (sum of isomers), the detected concentration was far below the HBM-I value of 300 ng/g lipid [75] as well as for the BE value of 190,000 ng/g lipid [74].

Dieldrin and β-HCH levels detected in the pooled sample were in the lower range compared to results from the WHO study (2017) [70] and/or of other European studies [76], and below the German reference value of 70 ng/g lipid.

Dioxins and furans were detected in a concentration of 0.0032 ng/g lipid WHO-PCDD/F-toxic equivalent (TEQ WHO-05) resp. 0.0021 ng/g lipid WHO-PCDD/F-toxic equivalent (TEQ WHO-22) in the pooled breast milk sample and were thus below the levels identified in the WHO study of 2000–2012 [74], in the WHO study of 2007 [70] as well as in other European studies [71]. The detected concentration was also below the mean level for Austria, with 0.011 ng/g lipid in the WHO study in 1992/93. Polychlorinated biphenyls were detected at a concentration of 0.0023 ng/g lipid WHO-PCB-TEQ (WHO-05) resp. 0.0012 ng/g lipid WHO-PCB-TEQ (WHO-22). The PCDD/F-PCB-TEQ in the present sample was 0.0033 ng/g (WHO-22), which is 16.5 times higher than the BE value (PCDD/F-PCB-TEQ) of 0.0002 ng/g lipid [74].

The re-evaluation of the WHO toxic equivalency factors (TEFs) for chlorinated dioxin-like compounds [77] leads to lower TEQs WHO-22 compared to TEQs WHO-05 in the pooled breast milk sample of this study, which is also shown with the data from other international investigations.

Aldrin, endrin, endosulfan, toxaphene, mirex, hexabromobiphenyl, pentachlorobenzene and chlordecone were not detected in the pooled breast milk sample above the respective detection limits. Similarly, with the exception of PFOS and PFOA, none of the PFASs tested for were detected.

#### 3.3.2. Declining Trends of POPs in Europe

Austria already participated in two out of the so-far five WHO breast milk surveys in the past where PCDD/Fs (1987/88 and 1992/93), dioxin-like PCBs and indicator PCBs (1992/93) were investigated. The results of these studies were published in the first and the second GMR [70,71]. Including the current data, there is a clear decreasing trend of the PCDD/F and PCB exposure in breast milk in Austria and other European countries since the 1980s [59]. However, it should be mentioned that the TWI value was reduced sevenfold by EFSA in 2018 due to new epidemiological and animal toxicity data availability. To protect us from dietary exposure to dioxins and dioxin-like PCBs, the TWI value of 2 pg/kg bw should not be exceeded in adolescents, adults and elderly people [78]. The sum of the substances of the DDT group in Austria with 120 ng/g lipid was quite comparable with other European countries in 2006/07 (range 33–156 ng/g lipid) [70]. Time trends show decreasing levels of DDT and its metabolites in the European region [59]. Furthermore, breast milk levels of the chlordane group, dieldrin and β-HCH are within the concentration ranges of the European region, for which declining trends were observed as well [59]. HCB, detected at a concentration of 14,9 ng/g lipid in this study, was within the range of European countries with 2.7–16.9 ng/g lipid in 2007 [70] and below the average level of 33 ng/g lipid reported more recently by WHO/UNEP (2021) [59]. However, while a few studies showed exceptionally high HCB levels in Europe, since they were collected nearby in either heavy industrial or agricultural areas, a decreasing trend of HCB averages was reported in Europe from 1972 to 2013 [59].

## 4. Conclusions

This study shows—in line with other European studies and studies performed under the GMP of the Stockholm Convention—that the overall exposure to POPs has decreased, which clearly demonstrates the effectiveness of the outlined convention. However, new scientific evidence on the harmful effects of chemicals, particularly PBDEs and PFASs, shows that these chemicals are far more toxic than previously thought, including, e.g., adverse cognitive outcomes among children associated with early life exposure to penta-BDE mixtures. Since 2020, the risk to the immune system from the sum of PFOAs, PFNAs, PFHxSs and PFOSs has been considered particularly critical since infants with elevated blood levels of these four PFASs have often been found to have a reduced immune response after certain vaccinations. The TWI of 4.4 ng/kg bw should therefore not be exceeded in mothers to protect the breastfeeding infant from excessive PFAS exposure via breast milk and associated potential health risks.

However, although harmful substances may be present in breast milk, the health benefits of breastfeeding, such as reduced infant mortality and health benefits in adulthood, outweigh the risks, and exposure is not necessarily related to health risks. Nevertheless, exposure monitoring is important for the assessment of potential risks.

Obviously, prenatal exposure to PBDEs (i.e., in maternal blood and placenta) has a stronger effect on potential adverse birth outcomes than postnatal exposure and, therefore, it is more suitable for epidemiological studies investigating birth outcomes, but human breast milk is more easily accessible in comparison. Looking at adverse birth outcomes, the results of epidemiological studies using human breast milk can slightly vary compared to studies that use blood or tissue exposure levels. However, exposure assessment in breast milk can provide valuable information about potential prenatal as well as postnatal exposure, as higher breast milk exposure correlates with higher prenatal exposures in mothers.

Besides the monitoring of the current POPs, it is also important to keep in mind that possible substitution substances that have POP-like characteristics may be of concern as well. There are numerous examples of this in recent history. For example, BDE-209 was used to replace the penta- and octa-BDE compounds, and PFHxS was used as a substitute for PFOSs as well as PFNAs for PFOAs. These are examples of so-called regrettable substitutions. Efforts within the European Green Deal and the Chemical Strategy for Sustainability aim to ensure better chemical safety. Further, the universal PFAS restriction is intended to reduce the risks of this group of substances. The results of this study also support the conclusions of the POPs Review Committee that all efforts should be made to minimise the exposure to POPs in order to protect the health of our present and future generations.

## Figures and Tables

**Table 1:** Description of the study population groups of the pilot study (2013), the follow-up study (2014–2016) and the pooled sample investigated in the framework of the WHO/UNEP breast milk monitoring programme.

	Individual Breast Milk Samples			WHO/UNEP Pooled Sample
Sampling Period	2013		2014–2016	2014–2016
Substance groups (number of substances analysed)	PBDE (20)	PFAS (14)	PFAS (25)	POPs
Number of samples	18	21	40	1 pooled sample (from *n* = 34)
Age of the mother in years (mean ± SD)	22–40 (30 ± 6)	22–40 (31 ± 5)	21–36 (29 ± 4)	21–36 (28 ± 4)
Body weight after delivery in kg (mean ± SD)	51–96 (68 ± 12)	51–96 (68 ± 11)	-	-
Birth weight newborn in g (mean ± SD)	2800–4150 (3595 ± 361)	2800–4150 (3571 ± 360)	-	-

Abbreviation: SD = standard deviation.

**Table 2:** Results of polybrominated diphenyl ethers (PBDEs) in breast milk samples (in ng/g lipid and in ng/g breast milk) of 18 Austrian women sampled in 2013 (ranges, medians) and results from the Austrian pooled WHO/UNEP monitoring programme breast milk sample (in ng/g lipid). Table also includes results of calculated daily PBDE intakes (average and high intakes) via breast milk consumption in infants based on breast milk samples from 18 Austrian women sampled in 2013 (in ng/kg bodyweight/d) and results of daily PBDE intakes (average and high intakes) from other European studies listed in EFSA (2011) [21].

PBDE Congener Concentrations in Breast Milk from Austrian Women from 2013 (Pilot Study)							WHO Pooled Sample from 2014–2016 ^1^	Daily Intake via Breast Milk Consumption [ng/kg bw/d] ^2^					
			[ng/g lipid]		[ng/g breast milk]		[ng/g lipid]	Average intake (present study)		EFSA ^3^ Average intake	High intake (present study)		EFSA High intake
**Substance**	*n*	*n* < LOD	range	median	range	median		range	median (mean)		range	median (mean)	range
**BDE-15**	-	-	-	-	-	-	0.02	-	-	-	-	-	-
**BDE-17**	-	-	-	-	-	-	0.0017	-	-	-	-	-	-
**BDE-28**	18	5	n.d.–0.38	0.048	n.d.–0.012	<LOQ	0.0256	0.0–1.57	0.17 (0.60)	<0.05–1.38	0.0–2.36	0.26 (0.90)	<0.07–2.06
**BDE-47**	18	6	n.d.–6.0	0.32	n.d.–0.20	<LOQ	0.412	0.0–26.2	1.84 (6.11)	0.64–13.77	0.0–39.3	2.75 (9.17)	0.96–20.64
**BDE-49**	18	11	n.d.–0.67	n.d.	n.d.–0.021	n.d.	0.01	0.0–2.75	0.0 (0.32)	-	0.0–4.13	0.0 (0.48)	-
**BDE-66**	18	11	n.d.–0.48	n.d.	n.d.–0.015	n.d.	<LOQ	0.0–1.97	0.0 (0.25)	-	0.0–2.95	0.0 (0.37)	-
**BDE-75**	-	-	-	-	-	-	<LOQ	-	-	-	-	-	-
**BDE-77**	18	12	n.d.–0.38	n.d.	n.d.–0.012	n.d.	<LOQ	0.0–1.57	0.0 (0.17)	-	0.0–2.36	0.0 (0.25)	-
**BDE-85**	18	18	n.d.	n.d.	n.d.	n.d.	-	0.0	0.0 (0.0)	-	0.0	0.0 (0.0)	-
**BDE-99**	18	6	n.d.–2.4	0.153	n.d.–0.11	<LOQ	0.128	0.0–14.43	0.79 (3.05)	<0.14–5.05	0.0–21.64	1.18 (4.57)	<0.14–7.57
**BDE-100**	18	4	n.d.–1.1	0.16	n.d.–0.034	0.0065	0.126	0.0–4.46	0.85 (1.23)	0.18–4.59	0.0–6.69	1.28 (1.85)	0.28–6.88
**BDE-118**	18	12	n.d.–0.38	n.d.	n.d.–0.012	n.d.	-	0.0–1.57	0.0 (0.18)	-	0.0–2.36	0.0 (0.27)	-
**BDE-119**	-	-	-	-	-	-	0.01	-	-	-	-	-	-
**BDE-126**	18	17	n.d.–0.024	n.d.	n.d.–0.0011	n.d.	-	0.0–0.14	0.0 (0.01)	-	0.0–0.22	0.0 (0.01)	-
**BDE-138**	-	-	-	-	-	-	0.0042	-	-	-	-	-	-
**BDE-139**	18	14	n.d.–0.25	n.d.	n.d.–0.0079	n.d.	-	0.0–1.04	0.0 (0.10)	-	0.0–1.55	0.0 (0.15)	-
**BDE-153**	18	2	n.d.–0.86	0.23	n.d.–0.028	0.011	0.548	0.0–5.51	1.44 (1.54)	0.46–11.02	0.0–5.51	2.16 (2.31)	0.69–16.51
**BDE-154**	18	12	n.d.–1.3	n.d.	n.d.–0.041	n.d.	0.0124	0.0–5.38	0.0 (0.57)	<0.05–2.30	0.0–8.07	0.0 (0.85)	<0.07–3.44
**BDE-181**	18	18	n.d.	n.d.	n.d.	n.d.	-	0.0	0.0 (0.0)	-	0.0	0.0 (0.0)	-
**BDE-183**	18	3	n.d.–0.37	0.054	n.d.–0.012	<LOQ	0.05	0.0–1.57	0.25 (0.52)	0.23–1.38	0.0–2.36	0.37 (0.78)	0.34–2.06
**BDE-190**	-	-	-	-	-	-	<LOQ	-	-	-	-	-	-
**BDE-196**	18	13	n.d.–0.21	n.d.	n.d.–0.011	n.d.	-	0.0–1.44	0.0 (0.19)	-	0.0–2.16	0.0 (0.29)	-
**BDE-197**	18	1	n.d.–0.39	0.095	n.d.–0.020	<LOQ	-	0.0–2.62	0.70 (0.75)	-	0.0–3.93	1.04 (1.13)	-
**BDE-203**	18	13	n.d.–0.35	n.d.	n.d.–0.018	n.d.	1.35	0.0–2.36	0.0 (0.30)	-	0.0–3.54	0.0 (0.45)	-
**BDE-206**	-	-	-	-	-	-	1.35	-	-	-	-	-	-
**BDE-207**	18	9	n.d.–2.5	0.035	n.d.–0.13	<LOQ	0.24	0.0–17.1	1.93 (3.51)	-	0.0–25.6	2.90 (5.27)	-
**BDE-208**	-	-	-	-	-	-	0.1	-	-	-	-	-	-
**BDE-209**	18	8	n.d.–43	9.5	n.d.–2.2	<LOQ	-	0.0–289	57.1 (70.4)	0.96–13.31	0.0–433	85.6 (106)	1.44–19.95
**Sum 8 BDE ^4^**	18	1	n.d.–12.5	1.06	n.d.–0.39	0.056	-	0.0–51.5	7.32 (13.34)	-	0.0–77.3	10.98 (20.02)	-
**Sum PBDE**	18	0	0.055–52	11	0.002–2.53	0.47	4.38 ^5^	0.28–332	61.90 (89.78)	-	0.41–497	92.84 (135)	-
***Technical mixtures***													
**TetraBDE ^6^**	18	6	n.d.–0.238	0.0169	n.d.–7.53	0.423	-	-	-	-	-	-	-
**PentaBDE ^7^**	18	3	n.d.–0.134	0.0135	n.d.–3.88	-	-	-	-	-	-	-	-
**HexaBDE ^8^**	18	2	n.d.–0.0759	0.0125	n.d.–2.41	-	-	-	-	-	-	-	-
**HeptaBDE ^9^**	18	3	n.d.–0.012	0.0019	n.d.–0.37	-	-	-	-	-	-	-	-
**OctaBDE ^10^**	18	1	n.d.–0.044	0.0063	n.d.–0.85	-	-	-	-	-	-	-	-

Abbreviations: LOD: limit of detection; LOQ: limit of quantification; *n*: sample size; n.d.: not detected. ^1^ pooled breast milk sample from 34 Austrian women aged 21–36 years from 2014 to 2016. ^2^ daily intakes were calculated based on results of PBDE congener concentrations in individual breast milk samples of this study (*n* = 18) according to the calculation procedure published in EFSA (2011) [21] and considering the following assumptions: 800 mL breast milk consumption per day for average intake, 1200 mL breast milk consumption per day for high intake and 6.1 kg infant bodyweight. ^3^ Source: EFSA (2011) [21]. ^4^ sum of BDE-28, BDE-47, BDE-66, BDE-85, BDE-99, BDE-100, BDE-153 and BDE-154. ^5^ sum of BDE-15, BDE-17, BDE-28, BDE-47, BDE-49, BDE-66, BDE-75, BDE-99, BDE-100, BDE-119, BDE-138, BDE-153, BDE-154, BDE-183, BDE-190, BDE-203, BDE-204, BDE-207 and BDE208. ^6^ TetraBDE: sum of BDE-47, BDE-49, BDE-66 and BDE-77. ^7^ PentaBDE: sum of BDE-85, BDE-99, BDE-100, BDE-118 and BDE-126. ^8^ HexaBDE: sum of BDE-139, BDE-153 and BDE-154. ^9^ HeptaBDE: sum of BDE-181 and BDE-183. ^10^ OctaBDE: sum of BDE-196, BDE-197 and BDE-203.

**Table 3:** Concentrations of PFOA and PFOS in breast milk samples from Austrian women from 2013 (ranges, medians, 95th percentiles in ng/L) and calculated average and high weekly intakes via breast milk consumption in infants (ranges, medians, means in ng/kg bodyweight/week).

PFAS Concentrations in Breast Milk [ng/L]						Weekly Intake via Breast Milk Consumption [ng/kg bw/Week] ^a^			
						Average intake		High intake	
Substance	*n*	*n* < LOD	range	median	P95	range	median (mean)	range	median (mean)
Pilot study (2013)									
PFOA	21	11	n.d.–83	n.d.	81	0.0–76	0.0 (13)	0.0–114	0.0 (20)
PFOS	21	0	58–310	110	300	53–285	101 (116)	80–427	151 (174)
Sum 4 PFAS ^b^	21	0	69–310	120	308	63–285	110 (129)	95–427	165 (194)
Follow-up study (2014–2016)									
									
PFBA	40	36	n.d.–<LOQ	n.d.	<LOQ				
PFBS	40	39	n.d.–8.1	n.d.	n.d.				
PFPeS	40	36	n.d.–18	n.d.	<LOQ				
PFHxA	40	27	n.d.–28	n.d.	16				
PFHxS	40	0	<LOQ–9.7	<LOQ	6.0	1.9–8.9	1.9 (2.5)	2.8–13	2.8 (3.7)
PFHpA	40	30	n.d.–<LOQ	n.d.	<LOQ				
PFOA	40	0	<LOQ–91	25	75	4.1–84	22 (28)	6.3–125	34 (41)
PFOS	40	1	n.d.–55	12	39	0.0–50	11 (14)	0.0–76	17 (20)
PFNA	40	6	n.d.–15	<LOQ	8.0	0.0–14	2.1 (3.0)	0.0–21	3.2 (4.5)
PFDA	40	22	n.d.–8.7	n.d.	4.6				
PFDS	40	39	n.d.–<LOQ	n.d.	n.d.				
PFUnDA	39	36	n.d.–<LOQ	n.d.	<LOQ				
PFDoDA	39	37	n.d.–<LOQ	n.d.	<LOQ				
PFTrDA	40	39	n.d.–<LOQ	n.d.	n.d.				
PFTeDA	39	37	n.d.–<LOQ	n.d.	<LOQ				
6:2-FTS	40	31	n.d.–<LOQ	n.d.	<LOQ				
DONA	40	31	n.d.–9.6	n.d.	<LOQ				
6:2 Cl-PFESA	40	39	n.d.–5.5	n.d.	n.d.				
GenX	40	39	n.d.–14	n.d.	n.d.				
Sum 4 PFAS ^b^	40	0	14–124	44	119	13–114	40 (47)	20–170	61 (70)
									
WHO/UNEP monitoring programme (in ng/g)									
PFOS	1	1	0.025 ^c^						
PFOA	1	1	0.045 ^c^						

Abbreviations: LOD = limit of detection, LOQ = limit of quantification, *n* = sample size, P95 = 95th percentile, bw = body weight, n.d. = not detected. ^a^ daily intakes were calculated based on results of PFAS concentrations in breast milk samples of this study according to the calculation procedure published in EFSA (2011) and considering the following assumptions: 800 mL breast milk consumption per day for average intake, 1200 mL breast milk consumption per day for high intake and 6.1 kg infant bodyweight. Daily intakes were calculated for weekly intake for comparison with EFSA tolerable weekly intakes (TWI), where available. ^b^ Sum of PFOS, PFOA, PFNA and PFHxS. ^c^ provided in ng/g milk.

## Data Availability

Individual data are unavailable due to the privacy concerns outlined within the informed consent forms.

## Supplementary Materials

The following supporting information can be downloaded at: https://www.mdpi.com/article/10.3390/jox14010015/s1, Table S1: Investigated substances, limits of detection (LODs) and limits of quantification (LOQs); Table S2: Recovery rates for PBDEs and PFASs; Table S3: Assignment of the native PBDE standards to the 13C-labelled standards for recovery correction; Table S4: Correlations (Spearman’s) between levels of PBDE congeners in breast milk (n = 18) of the pilot study (2013); Table S5: Results on PBDE congeners and total PBDE concentrations (ranges, means, medians in ng/g lipid; detection rates in %) from selected studies in European populations; Table S6: Results on PFOA and PFOS concentrations (ranges, means, medians; detection rates in %) from selected studies in European populations; Table S7: POPs detected in the pooled Austrian sample within WHO/UNEP. Refs [79,80,81,82,83,84,85,86,87,88,89,90,91,92,93,94,95,96,97,98,99,100,101,102] are cited in Supplementary Materials.

## References

[1] Grummer-Strawn L.M., Rollins N. (2015). Summarising the health effects of breastfeeding. Acta Paediatr..

[2] WHO (2022). Breastfeeding.

[3] ECHA (2022). Understanding POPs.

[4] UNEP (2022). Persistent Organic Pollutants (POPs).

[5] Fenton S.E., Ducatman A., Boobis A., DeWitt J.C., Lau C., Ng C., Smith J.S., Roberts S.M. (2021). Per- and Polyfluoroalkyl Substance Toxicity and Human Health Review: Current State of Knowledge and Strategies for Informing Future Research. Environ. Toxicol. Chem..

[6] Croes K., Colles A., Koppen G., Govarts E., Bruckers L., van de Mieroop E., Nelen V., Covaci A., Dirtu A.C., Thomsen C. (2012). Persistent organic pollutants (POPs) in human milk: A biomonitoring study in rural areas of Flanders (Belgium). Chemosphere.

[7] UNEP (2013). Results of the Global Survey on Concentrations in Human Milk of Persistent Organic Pollutants by the United Nations Environment Programme and the World Health Organization.

[8] Frederiksen M., Vorkamp K., Thomsen M., Knudsen L.E. (2009). Human internal and external exposure to PBDEs—A review of levels and sources. Int. J. Hyg. Environ. Health.

[9] La Guardia M.J., Hale R.C., Harvey E. (2006). Detailed polybrominated diphenyl ether (PBDE) congener composition of the widely used penta-, octa-, and deca-PBDE technical flame-retardant mixtures. Environ. Sci. Technol..

[10] Eriksson P., Viberg H., Jakobsson E., Orn U., Fredriksson A. (2002). A brominated flame retardant, 2,2′,4,4′,5-pentabromodiphenyl ether: Uptake, retention, and induction of neurobehavioral alterations in mice during a critical phase of neonatal brain development. Toxicol. Sci..

[11] Akortia E., Okonkwo J.O., Lupankwa M., Osae S.D., Daso A.P., Olukunle O.I., Chaudhary A. (2016). A review of sources, levels, and toxicity of polybrominated diphenyl ethers (PBDEs) and their transformation and transport in various environmental compartments. Environ. Rev..

[12] Besis A., Samara C. (2012). Polybrominated diphenyl ethers (PBDEs) in the indoor and outdoor environments—A review on occurrence and human exposure. Environ. Pollut..

[13] Fromme H., Becher G., Hilger B., Völkel W. (2016). Brominated flame retardants—Exposure and risk assessment for the general population. Int. J. Hyg. Environ. Health.

[14] Zhang Y., Wang W., Song J., Ren Z., Yuan H., Yan H., Zhang J., Pei Z., He Z. (2016). Environmental Characteristics of Polybrominated Diphenyl Ethers in Marine System, with Emphasis on Marine Organisms and Sediments. Biomed. Res. Int..

[15] Jakobsson K., Fång J., Athanasiadou M., Rignell-Hydbom A., Bergman A. (2012). Polybrominated diphenyl ethers in maternal serum, umbilical cord serum, colostrum and mature breast milk. Insights from a pilot study and the literature. Environ. Int..

[16] Klinčić D., Dvoršćak M., Jagić K., Mendaš G., Herceg Romanić S. (2020). Levels and distribution of polybrominated diphenyl ethers in humans and environmental compartments: A comprehensive review of the last five years of research. Environ. Sci. Pollut. Res. Int..

[17] Mannetje A., Coakley J., Mueller J.F., Harden F., Toms L.-M., Douwes J. (2012). Partitioning of persistent organic pollutants (POPs) between human serum and breast milk: A literature review. Chemosphere.

[18] Tang J., Zhai J.X. (2017). Distribution of polybrominated diphenyl ethers in breast milk, cord blood and placentas: A systematic review. Environ. Sci. Pollut. Res. Int..

[19] Gill U., Chu I., Ryan J.J., Feeley M. (2004). Polybrominated diphenyl ethers: Human tissue levels and toxicology. Rev. Environ. Contam. Toxicol..

[20] Linares V., Bellés M., Domingo J.L. (2015). Human exposure to PBDE and critical evaluation of health hazards. Arch. Toxicol..

[21] EFSA (2011). Scientific Opinion on Polybrominated Diphenyl Ethers (PBDEs) in Food. European Food Safety Authority. EFSA J..

[22] Stockholm Convention (2023). All POPs Listed in the Stockholm Convention.

[23] EFSA (2020). Scientific Opinion Risk to Human Health Related to the Presence of Perfluoroalkyl Substances in Food. Eur. Food Saf. Auth. J..

[24] OECD (2021). Reconciling Terminology of the Universe of Per- and Polyfluoroalkyl Substances: Recommendations and Practical Guidance; OECD Environment, Organisation for Economic Co-operation and Development. Health Saf. Publ. Ser. Risk Manag..

[25] OECD (2018). Toward a New Comprehensive Global Database of Per- and Polyfluoroalkyl Substances (PFASs): Summary Report on Updating the OECD, 2007 List of Per- and Polyfluoroalkyl Substances (PFASs).

[26] Buck R.C., Franklin J., Berger U., Conder J.M., Cousins I.T., Voogt P., de Jensen A.A., Kannan K., Mabury S.A., van Leeuwen S.P.J. (2011). Perfluoroalkyl and polyfluoroalkyl substances in the environment: Terminology, classification, and origins. Integr. Environ. Assess. Manag..

[27] EFSA (2008). Perfluorooctane sulfonate (PFOS), perfluorooctanoic acid (PFOA) and their salts. Scientific Opinion of the Panel on Contaminants in the Food chain. Eur. Food Saf. Auth. EFSA J..

[28] Haug L.S., Becher G., Knudsen L., Merlo D.F. (2011). Biomarkers of Exposure: Perfluoroalkyl Compounds. Biomarkers and Human Biomonitoring.

[29] Glüge J., Scheringer M., Cousins I.T., DeWitt J.C., Goldenman G., Herzke D., Lohmann R., Ng C.A., Trier X., Wang Z. (2020). An overview of the uses of per- and polyfluoroalkyl substances (PFAS). Environ. Sci. Process. Impacts.

[30] Stockholm Convention (2022). Chemicals Proposed for Listing under the Convention. http://www.pops.int/TheConvention/ThePOPs/ChemicalsProposedforListing/tabid/2510/Default.aspx.

[31] D’Hollander W., de Voogt P., De Coen W., Bervoets L. (2010). Perfluorinated substances in human food and other sources of human exposure. Rev. Environ. Contam. Toxicol..

[32] Olsen G.W., Burris J.M., Ehresman D.J., Froehlich J.W., Seacat A.M., Butenhoff J.L., Zobel L.R. (2007). Half-life of serum elimination of perfluorooctanesulfonate, perfluorohexanesulfonate, and perfluorooctanoate in retired fluorochemical production workers. Environ. Health Perspect..

[33] Lau C., Anitole K., Hodes C., Lai D., Pfahles-Hutchens A., Seed J. (2007). Perfluoroalkyl acids: A review of monitoring and toxicological findings. Toxicol. Sci..

[34] Miralles-Marco A., Harrad S. (2015). Perfluorooctane sulfonate: A review of human exposure, biomonitoring and the environmental forensics utility of its chirality and isomer distribution. Environ. Int..

[35] Harada K., Inoue K., Morikawa A., Yoshinaga T., Saito N., Koizumi A. (2005). Renal clearance of perfluorooctane sulfonate and perfluorooctanoate in humans and their species-specific excretion. Environ. Res..

[36] Hartmann C., Raffesberg W., Weiss S., Scharf S., Uhl M. (2017). Perfluoroalkylated substances in human urine: Results of a biomonitoring pilot study. Biomonitoring.

[37] Zhang Y., Beesoon S., Zhu L., Martin J.W. (2013). Biomonitoring of perfluoroalkyl acids in human urine and estimates of biological half-life. Environ. Sci. Technol..

[38] Antignac J.-P., Veyrand B., Kadar H., Marchand P., Oleko A., Le Bizec B., Vandentorren S. (2013). Occurrence of perfluorinated alkylated substances in breast milk of French women and relation with socio-demographical and clinical parameters: Results of the ELFE pilot study. Chemosphere.

[39] Awad R., Zhou Y., Nyberg E., Namazkar S., Yongning W., Xiao Q., Sun Y., Zhu Z., Bergman Å., Benskin J.P. (2020). Emerging per- and polyfluoroalkyl substances (PFAS) in human milk from Sweden and China. Environ. Sci. Process. Impacts.

[40] Fiedler H., Sadia M. (2021). Regional occurrence of perfluoroalkane substances in human milk for the global monitoring plan under the Stockholm Convention on Persistent Organic Pollutants during 2016-2019. Chemosphere.

[41] Zheng G., Schreder E., Dempsey J.C., Uding N., Chu V., Andres G., Sathyanarayana S., Salamova A. (2021). Per- and Polyfluoroalkyl Substances (PFAS) in Breast Milk: Concerning Trends for Current-Use PFAS. Environ. Sci. Technol..

[42] DeWitt J.C., Peden-Adams M.M., Keller J.M., Germolec D.R. (2012). Immunotoxicity of perfluorinated compounds: Recent developments. Toxicol. Pathol..

[43] EFSA (2018). Risk to human health related to the presence of perfluorooctane sulfonic acid and perfluorooctanoic acid in food. Eur. Food Saf. Auth. J..

[44] UNEP (2017). Global Monitoring Plan on Persistent Organic Pollutants: Guidelines for Organization, Sampling and Analysis of Human Milk on Persistent Organic Pollutants.

[45] U.S. EPA (2010). Method 1614A—Brominated Diphenyl Ethers in Water, Soil, Sediment, and Tissue by HRGC/HRMS: Engineering and Analysis Division (4303T).

[46] Kuklenyik Z., Reich J.A., Tully J.S., Needham L.L., Calafat A.M. (2004). Automated solid-phase extraction and measurement of perfluorinated organic acids and amides in human serum and milk. Environ. Sci. Technol..

[47] (2008). Chemical Analysis—Decision Limit, Detection Limit and Determination Limit under Repeatability Conditions—Terms, Methods.

[48] Kärrman A., Ericson I., van Bavel B., Darnerud P.O., Aune M., Glynn A., Lignell S., Lindström G. (2007). Exposure of perfluorinated chemicals through lactation: Levels of matched human milk and serum and a temporal trend, 1996–2004, in Sweden. Environ. Health Perspect..

[49] Main K.M., Kiviranta H., Virtanen H.E., Sundqvist E., Tuomisto J.T., Tuomisto J., Vartiainen T., Skakkebaek N.E., Toppari J. (2007). Flame retardants in placenta and breast milk and cryptorchidism in newborn boys. Environ. Health Perspect..

[50] Polder A., Thomsen C., Lindström G., Løken K.B., Skaare J.U. (2008). Levels and temporal trends of chlorinated pesticides, polychlorinated biphenyls and brominated flame retardants in individual human breast milk samples from Northern and Southern Norway. Chemosphere.

[51] Raab U., Preiss U., Albrecht M., Shahin N., Parlar H., Fromme H. (2008). Concentrations of polybrominated diphenyl ethers, organochlorine compounds and nitro musks in mother’s milk from Germany (Bavaria). Chemosphere.

[52] Lignell S., Aune M., Darnerud P.O., Cnattingius S., Glynn A. (2009). Persistent organochlorine and organobromine compounds in mother’s milk from Sweden 1996-2006: Compound-specific temporal trends. Environ. Res..

[53] Jaraczewska K., Lulek J., Covaci A., Voorspoels S., Kaluba-Skotarczak A., Drews K., Schepens P. (2006). Distribution of polychlorinated biphenyls, organochlorine pesticides and polybrominated diphenyl ethers in human umbilical cord serum, maternal serum and milk from Wielkopolska region, Poland. Sci. Total Environ..

[54] Antignac J.-P., Cariou R., Zalko D., Berrebi A., Cravedi J.-P., Maume D., Marchand P., Monteau F., Riu A., Andre F. (2009). Exposure assessment of French women and their newborn to brominated flame retardants: Determination of tri- to deca- polybromodiphenylethers (PBDE) in maternal adipose tissue, serum, breast milk and cord serum. Environ. Pollut..

[55] Abdallah M.A.-E., Harrad S. (2014). Polybrominated diphenyl ethers in UK human milk: Implications for infant exposure and relationship to external exposure. Environ. Int..

[56] Bramwell L., Fernandes A., Rose M., Harrad S., Pless-Mulloli T. (2014). PBDEs and PBBs in human serum and breast milk from cohabiting UK couples. Chemosphere.

[57] Lee S., Kim S., Kim E., Lee I.-S., Choi G., Kim H.-J., Park J., Jae Lee J., Choi S., Young Kim S. (2013). Polybrominated diphenyl ethers (PBDEs) in breast milk of Korea in 2011: Current contamination, time course variation, influencing factors and health risks. Environ. Res..

[58] Daniels J.L., Pan I.-J., Jones R., Anderson S., Patterson D.G., Needham L.L., Sjödin A. (2010). Individual characteristics associated with PBDE levels in U.S. human milk samples. Environ. Health Perspect..

[59] WHO/UNEP (2021). Global Monitoring Plan for Persistent Organic Pollutants under the Stockholm Convention Article 16 on Effectiveness Evaluation—Third Regional Monitoring Report Western Europe and Others Group (WEOG) Region 2021.

[60] Zhao X., Peng S., Xiang Y., Yang Y., Li J., Shan Z., Teng W. (2017). Correlation between Prenatal Exposure to Polybrominated Diphenyl Ethers (PBDEs) and Infant Birth Outcomes: A Meta-Analysis and an Experimental Study. Int. J. Environ. Res. Public Health.

[61] Chao H.-R., Wang S.-L., Lee W.-J., Wang Y.-F., Päpke O. (2007). Levels of polybrominated diphenyl ethers (PBDEs) in breast milk from central Taiwan and their relation to infant birth outcome and maternal menstruation effects. Environ. Int..

[62] Miranda M.L., Anthopolos R., Wolkin A., Stapleton H.M. (2015). Associations of birth outcomes with maternal polybrominated diphenyl ethers and thyroid hormones during pregnancy. Environ. Int..

[63] Zhao Y., Song Q., Ge W., Jin Y., Chen S., Zhao Y., Xiao X., Zhang Y. (2019). Associations between in utero exposure to polybrominated diphenyl ethers, pathophysiological state of fetal growth and placental DNA methylation changes. Environ. Int..

[64] Costa L.G., Giordano G. (2011). Is decabromodiphenyl ether (BDE-209) a developmental neurotoxicant?. NeuroToxicology.

[65] Herbstman J.B., Mall J.K. (2014). Developmental Exposure to Polybrominated Diphenyl Ethers and Neurodevelopment. Curr. Environ. Health Rep..

[66] Poston R.G., Saha R.N. (2019). Epigenetic Effects of Polybrominated Diphenyl Ethers on Human Health. Int. J. Environ. Res. Public Health.

[67] Völkel W., Genzel-Boroviczény O., Demmelmair H., Gebauer C., Koletzko B., Twardella D., Raab U., Fromme H. (2008). Perfluorooctane sulphonate (PFOS) and perfluorooctanoic acid (PFOA) in human breast milk: Results of a pilot study. Int. J. Hyg. Environ. Health.

[68] Kärrman A., Domingo J.L., Llebaria X., Nadal M., Bigas E., van Bavel B., Lindström G. (2010). Biomonitoring perfluorinated compounds in Catalonia, Spain: Concentrations and trends in human liver and milk samples. Environ. Sci. Pollut. Res. Int..

[69] Guerranti C., Perra G., Corsolini S., Focardi S.E. (2013). Pilot study on levels of perfluorooctane sulfonic acid (PFOS) and perfluorooctanoic acid (PFOA) in selected foodstuffs and human milk from Italy. Food Chem..

[70] WHO/UNEP (2009). Global Monitoring Plan for Persistent Organic Pollutants under the Stockholm Convention Article 16 on Effectiveness Evaluation—First Regional Monitoring Report Western Europe and Others Group (WEOG) Region 2008.

[71] WHO/UNEP (2015). Global Monitoring Plan for Persistent Organic Pollutants under the Stockholm Convention Article 16 of Effectiveness Evaluation—2nd Regional Monitoring Report Western Europe and Others Group (WEOG) Region 2015.

[72] Fiedler H. (2023). Metadata Analysis of Persistent Organic Pollutants in National Pools of Human Milk in Support of the Stockholm Convention Implementation. Environ. Health.

[73] Umweltbundesamt (2009). Referenzwerte Für Organochlorpestizide und PCB in Frauenmilch.

[74] WHO (2015). Human Biomonitoring: Facts and Figures.

[75] Umweltbundesamt (2015). Factsheet HBM Value for HBCD.

[76] Gyllenhammar I., Aune M., Fridén U., Cantillana T., Bignert A., Lignell S., Glynn A. (2021). Are temporal trends of some persistent organochlorine and organobromine compounds in Swedish breast milk slowing down?. Environ. Res..

[77] DeVito M., Bokkers B., van Duursen M.B.M., van Ede K., Feeley M., Antunes Fernandes Gáspár E., Haws L., Kennedy S., Peterson R.E., Hoogenboom R. (2024). The 2022 world health organization reevaluation of human and mammalian toxic equivalency factors of polychlorinated dioxins, dibenzofurans and biphenyls. Regul. Toxicol. Pharmacol..

[78] EFSA (2018). Risk for animal and human health related to the presence of dioxins and dioxin-like PCBs in feed and food. Eur. Food Saf. Auth. J..

[79] Ingelido A.M., Ballard T., Dellatte E., Di Domenico A., Ferri F., Fulgenzi A.R., Herrmann T., Iacovella N., Miniero R., Päpke O. (2007). Polychlorinated biphenyls (PCBs) and polybrominated diphenyl ethers (PBDEs) in milk from Italian women living in Rome and Venice. Chemosphere.

[80] Kazda R., Hajšlová J., Poustka J., Čajka T. (2004). Determination of polybrominated diphenyl ethers in human milk samples in the Czech Republic: Comparative study of negative chemical ionization mass spectrometry and time-of-flight high-resolution mass spectrometry. Anal. Chim. Acta.

[81] Gómara B., Herrero L., Ramos J.J., Mateo J.R., Fernández M.A., García J.F., González M.J. (2007). Distribution of polybrominated diphenyl ethers in human umbilical cord serum, paternal serum, maternal serum, placentas, and breast milk from Madrid population, Spain. Environ. Sci. Technol..

[82] Thomsen C., Stigum H., Frøshaug M., Broadwell S.L., Becher G., Eggesbø M. (2010). Determinants of brominated flame retardants in breast milk from a large scale Norwegian study. Environ. Int..

[83] Bordajandi L.R., Abad E., González M.J. (2008). Occurrence of PCBs, PCDD/Fs, PBDEs and DDTs in Spanish breast milk: Enantiomeric fraction of chiral PCBs. Chemosphere.

[84] Gómara B., Herrero L., Pacepavicius G., Ohta S., Alaee M., González M.J. (2011). Occurrence of co-planar polybrominated/chlorinated biphenyls (PXBs), polybrominated diphenyl ethers (PBDEs) and polychlorinated biphenyls (PCBs) in breast milk of women from Spain. Chemosphere.

[85] Chovancová J., Čonka K., Kočan A., Sejáková Z.S. (2011). PCDD, PCDF, PCB and PBDE concentrations in breast milk of mothers residing in selected areas of Slovakia. Chemosphere.

[86] Fromme H., Fuchs V., Albrecht M., Aschenbrenner B., Röhl C., Janitzki N., Herber-Jonat S., Wöckner M., Völkel W., Flemmer A.W. (2022). Polychlorinated dioxins and dibenzofurans (PCDD/F), polybrominated dioxins and dibenzofurans (PBDD/F), polychlorinated biphenyls (PCB), polybrominated diphenyl ethers (PBDE), and per- and polyfluoroalkyl substances (PFAS) in German breast milk samples (LUPE 8). Sci. Total Environ..

[87] Roosens L., D’Hollander W., Bervoets L., Reynders H., van Campenhout K., Cornelis C., van den Heuvel R., Koppen G., Covaci A. (2010). Brominated flame retardants and perfluorinated chemicals, two groups of persistent contaminants in Belgian human blood and milk. Environ. Pollut..

[88] Raab U., Albrecht M., Preiss U., Völkel W., Schwegler U., Fromme H. (2013). Organochlorine compounds, nitro musks and perfluorinated substances in breast milk—Results from Bavarian Monitoring of Breast Milk 2007/8. Chemosphere.

[89] Llorca M., Farré M., Picó Y., Teijón M.L., Alvarez J.G., Barceló D. (2010). Infant exposure of perfluorinated compounds: Levels in breast milk and commercial baby food. Environ. Int..

[90] Barbarossa A., Masetti R., Gazzotti T., Zama D., Astolfi A., Veyrand B., Pession A., Pagliuca G. (2013). Perfluoroalkyl substances in human milk: A first survey in Italy. Environ. Int..

[91] Cariou R., Veyrand B., Yamada A., Berrebi A., Zalko D., Durand S., Pollono C., Marchand P., Leblanc J.-C., Antignac J.-P. (2015). Perfluoroalkyl acid (PFAA) levels and profiles in breast milk, maternal and cord serum of French women and their newborns. Environ. Int..

[92] Zeilmaker M.J., Moermond C., Brandon E., Hoogerhuis P., Razenberg L., Janssen M. (2020). Persistent Organic Pollutants in Human Milk in the Netherlands.

[93] Černá M., Grafnetterová A.P., Dvořáková D., Pulkrabová J., Malý M., Janoš T., Vodrážková N., Tupá Z., Puklová V. (2020). Biomonitoring of PFOA, PFOS and PFNA in human milk from Czech Republic, time trends and estimation of infant’s daily intake. Environ. Res..

[94] Abdallah M.A.-E., Wemken N., Drage D.S., Tlustos C., Cellarius C., Cleere K., Morrison J.J., Daly S., Coggins M.A., Harrad S. (2020). Concentrations of perfluoroalkyl substances in human milk from Ireland: Implications for adult and nursing infant exposure. Chemosphere.

[95] ATSDR (2018). Minimal Risk Levels (MRLs) List. Agency for Toxic Substances and Disease Registry, U.S. Department of Health & Human Services, USA. https://www.atsdr.cdc.gov/mrls/mrllist.asp.

[96] EFSA (European Food Safety Authority) (2007). Chlordane as undesirable substance in animal feed—Scientific Opinion of the Panel on Contaminants in the Food Chain. EFSA J..

[97] EFSA (European Food Safety Authority) (2005). Opinion of the Scientific Panel on contaminants in the food chain [CONTAM] related to aldrin and dieldrin as undesirable substance in animal feed. EFSA J..

[98] EFSA (European Food Safety Authority) (2006). Opinion of the Scientific Panel on contaminants in the food chain [CONTAM] related to DDT as an undesirable substance in animal feed. EFSA J..

[99] EFSA (European Food Safety Authority) (2007). Opinion of the Scientific Panel on contaminants in the food chain [CONTAM] related heptachlor as an undesirable substance in animal feed. EFSA J..

[100] WHO (2001). Pesticide Residues in Food—2000: Toxicological Evaluations. World Health Organization, Geneva, Switzerland..

[101] EFSA (2019). Risk assessment of chlorinated paraffins in feed and food. EFSA J..

[102] Health Canada (2007). Non-Carcinogen Tolerable Daily Intake (TDI) Values from Health Canada. http://www.popstoolkit.com/tools/HHRA/TDI_HealthCanada.aspx.

