# Supplementary material for: Persistent Organic Pollutants in Austrian Human Breast Milk Collected between 2013 and 2016
Source: J Xenobiot. 2024 Feb 7;14(1):247–66. doi: 10.3390/jox14010015 (PMC10885091; doi:10.3390/jox14010015)
Supplement: Supplementary file 1 [file jox-14-00015-s001.zip › jox-2758593-supplementary.pdf]

Supplementary information (SI)

Persistent Organic Pollutants in Austrian Human Breast Milk collected between 2013 and 2016

Table S1: Investigated substances, limits of detection (LOD) and limits of quantification (LOQ).

Substance	Abbreviation	CAS#	LOD	LOQ	Measured in		
					(1)	*(2)*	*(3)*
PBDE congeners			***[ng/g milk] (1)***	***[ng/g milk] (1) [ng/g fat] (3)***			
4,4'-Dibromodiphenyl ether	BDE-15	2050-47-7		0.00017 (3)			x
2,2',4-Tribromodiphenyl ether	BDE-17	147217-75-2		0.00092 (3)			x
2,4,4'-Tribromodiphenyl ether	BDE-28	41318-75-6	0.0000068-0.00035 (1)	0.0026-0.0088 (1); 0.00093 (3)	x		x
2,2',4,4'-Tetrabromodiphenyl ether[*,1]	BDE-47	5436-43-1	0.000018-0.0005 (1)	0.028-0.095 (1); 0.00056 (3)	x		x
2,2',4,5'-Tetrabromodiphenyl ether[*,1]	BDE-49	243982-82-3	0.000019-0.00044 (1)	0.00014-0.00088 (1); 0.00091 (3)	x		x
2,3',4,4'-Tetrabromodiphenyl ether[*,1]	BDE-66	189084-61-5	0.000028-0.0012 (1)	0.00029-0.0024 (1); 0.00084 (3)	x		x
2,4,4',6-Tetrabromodiphenyl ether	BDE-75	189084-63-7		0.00059 (3)			x
3,3',4,4'-Tetrabromodiphenyl ether[*,1]	BDE-77	93703-48-1	0.000014-0.0005 (1)	0.00011-0.001 (1); 0.00048 (3)	x		x
2,2',3,4,4'-Pentabromodiphenyl ether[*,1]	BDE-85	182346-21-0	0.00016-0.0061 (1)	0.0033-0.012 (1)	x		
2,2',4,4',5-Pentabromodiphenyl ether[*,1]	BDE-99	60348-60-9	0.000028-0.00097 (1)	0.012-0.042 (1); 0.00170 (3)	x		x
2,2',4,4',6-Pentrabromodiphenyl ether[*,1]	BDE-100	189084-64-8	0.000022-0.001 (1)	0.0034-0.011 (1); 0.00195 (3)	x		x
2,3',4,4',5-Pentabromodiphenyl ether[*,1]	BDE-118	446254-80-4	0.000049-0.0014 (1)	0.00075-0.0028 (1)	x		
2,3',4,4',6-Pentabromodiphenyl ether	BDE-119	189084-66-0		0.00145 (3)			x
3,3',4,4',5-Pentabromodiphenyl ether[*,1]	BDE-126	366791-32-4	0.00011-0.0041 (1)	0.0022-0.0082 (1)	x		
2,2',3,4,4',5',6-Heptabromodiphenyl ether	BDE-138	207122-16-5		0.00089 (3)			x
2,2',3,4,4',6-Hexabromodiphenyl ether[*,1]	BDE-139	446254-96-2	0.000066-0.0018 (1)	0.00058-0.0036 (1)	x		
2,2',4,4',5,5'-Hexabromodiphenyl ether[*,1]	BDE-153	68631-49-2	0.000044-0.0012 (1)	0.00042-0.0024 (1); 0.00062 (3)	x		x
2,2'4,4'5,6'-Hexabromodiphenyl ether[*,1]	BDE-154	207122-15-4	0.000028-0.0016 (1)	0.00032-0.0032 (1); 0.00060 (3)	x		x
2,2',3,4,4',5,6-Heptabromodiphenyl ether[*,1]	BDE-181	189084-67-1	0.000061-0.00053 (1)	0.00012-0.0011 (1)	x		
2,2',3,4,4',5',6-Heptabromodiphenyl ether[*,1]	BDE-183	207122-16-5	0.000052-0.00046 (1)	0.0038-0.013 (1); 0.00249 (3)	x		x
2,3,3',4,4',5,6-Heptabromodiphenyl ether	BDE-190	189084-68-2		0.00479			x
2,2',3,3',4,4',5,6'-Octabromodiphenyl ether	BDE-196	446255-39-6	0.000085-0.0012 (1)	0.0054-0.018 (1)	x		
2,2',3,3',4,4',6,6'-Octabromodiphenyl ether	BDE-197	117964-21-3	0.000094-0.0013 (1)	0.0042-0.014 (1)	x		
2,2',3,4,4',5,5',6-Octabromodiphenyl ether	BDE-203	337513-72-1	0.001-0.002 (1)	0.006-0.02 (1); 0.00187 (3)	x		x

Substance	Abbreviation	CAS#	LOD	LOQ	Measured in (1)	(2)	(3)
2,2',3,3',4,4',5,5',6-Nonabromodiphenyl ether	BDE-206	63387-28-0		0.0126 (3)			x
2,2',3,3',4,4',5,6,6'-Nonabromodiphenyl ether	BDE-207	437701-79-6	0.00027-0.004 (1)	0.059-0.2 (1); 0.0072 (3)	x		x
2,2',3,3',4,4',5,5',6,6'-decabromodiphenyl ether[*,1]	BDE-209	1163-19-5	0.0019-0.021 (1)	0.87-4.0 (1)	x		
PFAS			***[ng/l]***	***[ng/l]***			
perfluoro-1-butanesulfonate	PFBS	375-73-5	7.7 (1); 1.6 (2)	28 (1); 7.7 (2)	x	x	
perfluoro-n-butanoic acid	PFBA	375-22-4	1.4	7.1-7.2		x	
perfluoro-n-pentanoic acid	PFPeA	2706-90-3	1.1 (2); 0.01[11] (3)	5.7 (2)		x	x
perfluoro-n-pentane sulfonate	PFPeS	630402-22-1	1.7	7.9		x	
perfluoro-n-hexanoic acid	PFHxA	307-24-4	2.3 (1); 1.4 (2); 0.01[11] (3)	8.4 (1); 7.1 (2)	x	x	x
perfluoro-1-hexanesulfonate[4]	PFHxS	355-46-4	6.2 (1); 9.0 (2); 0.01[11] (3)	23 (1); 4.1 (2)	x	x	x
perfluoro-n-heptanoic acid	PFHpA	375-85-9	2.7 (1); 1.4 (2); 0.01[11] (3)	10 (1); 6.1 (2)	x	x	x
perfluoro-heptanesulfonate	PFHpS	375-92-8	13 (1); 1.9 (2)	47 (1); 9.0 (2)	x	x	
perfluorooctanoic acid[*,1]	PFOA	335-67-1	6.2 (1); 1.4-2.8 (2); 0.015[11] (3)	22 (1); 6.1-12 (2)	x	x	x
perfluorooctanesulphonate[*,2]	PFOS	1763-23-1	13 (1); 2.4-8.4 (2); 0.01[11] (3)	47 (1); 11-38	x	x	x
perfluoro-n-nonanoic acid	PFNA	375-95-1	3.9 (1); 1.1-3.6 (2); 0.01[11] (3)	15 (1); 4.6-16 (2)	x	x	x
perfluoro-n-nonane sulfonate	PFNS	98789-57-2	1.5	6.9		x	
perfluoro-n-decanoic acid	PFDA	335-76-2	2.4 (1); 1.1-6.6 (2); 0.01[11] (3)	9.0 (1); 4.3-26 (2)	x	x	x
perfluoro decanesulfonate	PFDS	67906-42-7	10 (1); 1.4 (2)	36 (1); 6.6 (2)	x	x	
perfluoro-n-undecanoic acid	PFUnDA	2058-94-8	7.2 (1); 1.3-2.9 (2); 0.01[11] (3)	27 (1); 6.3-15 (2)	x	x	x
perfluoro-n-dodecanoic acid	PFDoA	307-55-1	3.4 (1); 1.3-2.6 (2)	26 (1); 6.5-13 (2)	x	x	
perfluoro-n-tridecanoic acid	PFTrDA	72629-94-8	10 (1); 2.4 (2)	37 (1); 12 (2)	x	x	
perfluoro-n-tetradecanoic acid	PFTeDA	376-06-7	13 (1); 2.0-4.9 (2)	47 (1); 9.5-23 (2)	x	x	
N-ethyl-perfluoro-n-octane sulfonamido acetic acid	EtFOSAA	2991-50-6	1.2-4.0	6.1-20		x	
4:2 fluorotelomer sulfonate	4:2 FTSA	757124-72-4	1.3	6.0		x	
6:2 fluorotelomer sulfonate	6:2 FTSA	27619-97-2	2.4	11		x	
8:2 fluorotelomer sulfonate	8:2 FTSA	39108-34-4	8.1	37		x	
dodecalfluoro-3H-4,8-dioxanonanoate	DONA	919005-14-4	1.2	5.8		x	
6:2 chlorinated polyfluorinated ether sulfonate	6:2 Cl-PFESA (F-53B)	73606-19-6	1.1	5.1		x	
hexafluoropropylene oxide-dimer acid	HFPO-DA (GenX)	13252-13-6	6.0	28-30		x	
Persistent organic pollutants (POPs)			***[ng/g] lipid***	***[ng/g] lipid***			
Aldrin[*,1]		309-00-2	0.5				x
Chlordane[*,1,5]		see footnote	0.5				x
Dieldrin[*,1]		60-57-1	0.5				x
DDT[*,2,6]		see footnote	0.5				x

Substance	Abbreviation	CAS#	LOD	LOQ	Measured in		
					(1)	*(2)*	*(3)*
Endrin[*,1,7]		see footnote	0.5				x
Heptachlor[*,1,8]		see footnote	0.5				x
Hexachlorobenzene[*,1,3]	HCB	118-74-1	0.5				x
alpha-Hexachlorocyclohexane[*,1]	α-HCH	319-84-6	0.5				x
beta-Hexachlorocyclohexane[*,1]	β-HCH	319-85-7	0.5				x
gamma-Hexachlorocyclohexane[*,1]	γ-HCH	58-89-9	0.5				x
Endosulfan[*,1,9]		see footnote	0.5				x
Toxaphene (Parlar) [*,1,10]		see footnote	0.5				x
Mirex[*,1]		2385-85-5	0.5				x
Hexabromobiphenyl[*,1]		36255-01-8	0.5				x
Pentachlorobenzene[*,1,3]		608-93-5	0.5				x
Chlordecone[*,1]		143-50-0	0.5				x
alpha-hexabromocyclododecane[*,1]	α-HBCD	134237-50-6	0.1				x
beta- hexabromocyclododecane[*,1]	β-HBCD	134237-51-7	0.1				x
gamma-hexabromocyclododecane[*,1]	γ-HBCD	134237-52-8	0.1				x
Persistent organic pollutants (POPs)				***[pg/g]***			
2,3,7,8-Tetrachlorodibenzofuran[*,3]	2,3,7,8-TCDF	51207-31-9		0.006			x
1,2,3,7,8-Pentachlorodibenzofuran[*,3]	1,2,3,7,8-PeCDF	57117-41-6		0.009			x
2,3,4,7,8-Pentachlorodibenzofuran[*,3]	2,3,4,7,8-PeCDF	57117-31-4		0.01			x
1,2,3,4,7,8-Hexachlorodibenzofuran[*,3]	1,2,3,4,7,8-HxCDF	70648-26-9		0.02			x
1,2,3,6,7,8-Hexachlorodibenzofuran[*,3]	1,2,3,6,7,8-HxCDF	57117-44-9		0.02			x
2,3,4,6,7,8-Hexachlorodibenzofuran[*,3]	2,3,4,6,7,8-HxCDF	60851-34-5		0.02			x
1,2,3,7,8,9-Hexachlorodibenzofuran[*,3]	1,2,3,7,8,9-HxCDF	72918-21-9		0.03			x
1,2,3,4,6,7,8-Heptachlorodibenzofuran[*,3]	1,2,3,4,6,7,8-HpCDF	67562-39-4		0.007			x
1,2,3,4,7,8,9-Heptachlorodibenzofuran[*,3]	1,2,3,4,7,8,9-HpCDF	55673-89-7		0.009			x
Octachlorodibenzofuran[*,3]	OCDF	39001-02-0		0.003			x
2,3,7,8-Tetrachlorodibenzo-p-dioxin[*,3]	2,3,7,8-TCDD	1746-01-6		0.005			x
1,2,3,7,8-Pentachlorodibenzo-p-dioxin[*,3]	1,2,3,7,8-PeCDD	40321-76-4		0.03			x
1,2,3,4,7,8-Hexachlorodibenzo-p-dioxin[*,3]	1,2,3,4,7,8-HxCDD	39227-28-6		0.03			x
1,2,3,6,7,8-Hexachlorodibenzo-p-dioxin[*,3]	1,2,3,6,7,8-HxCDD	57653-85-7		0.03			x
1,2,3,7,8,9-Hexachlorodibenzo-p-dioxin[*,3]	1,2,3,7,8,9-HxCDD	19408-74-3		0.03			x
1,2,3,4,6,7,8-Heptachlorodibenzo-p-dioxin[*,3]	1,2,3,4,6,7,8-HpCDD	35822-46-9		0.007			x
Octachlorodibenzo-p-dioxin[*,3]	OCDD	3268-87-9		0.02			x
Persistent organic pollutants (POPs)				***[ng/g]***			
2,4,4'-Trichlorobiphenyl[*,1]	PCB 28	7012-37-5		0.002			x
2,2',5,5'-Tetrachlorobiphenyl[*,1]	PCB 52	35693-99-3		0.006			x
3,3',4,4'-Tetrachlorobiphenyl[*,1]	PCB 77	32598-13-3		0.0000001			x
3,4,4',5-Tetrachlorobiphenyl[*,1]	PCB 81	70362-50-4		0.0000001			x
2,2',4,5,5'-Pentachlorobiphenyl[*,1]	PCB 101	37680-73-2		0.01			x

Substance	Abbreviation	CAS#	LOD	LOQ	Measured in		
					(1)	*(2)*	*(3)*
2,3,3',4,4'-Pentachlorobiphenyl[*,1]	PCB 105	32598-14-4		0.00002			x
2,3,4,4',5-Pentachlorobiphenyl[*,1]	PCB 114	74472-37-0		0.00001			x
2,3',4,4',5-Pentachlorobiphenyl[*,1]	PCB 118	31508-00-6		0.00001			x
2,3',4,4',5'-Pentachlorobiphenyl[*,1]	PCB 123	65510-44-3		0.00001			x
3,3',4,4',5-Pentachlorobiphenyl[*,1]	PCB 126	57465-28-8		0.0000003			x
2,2',3,4,4',5'-Hexachlorobiphenyl[*,1]	PCB 138	35065-28-2		0.02			x
2,2',4,4',5,5'-Hexachlorobiphenyl[*,1]	PCB 153	35065-27-1		0.02			x
2,3,3',4,4',5-Hexachlorobiphenyl[*,1]	PCB 156	38380-08-4		0.00002			x
2,3,3',4,4',5'-Hexachlorobiphenyl[*,1]	PCB 157	69782-90-7		0.00002			x
2,3',4,4',5,5'-Hexachlorobiphenyl[*,1]	PCB 167	52663-72-6		0.00002			x
3,3',4,4',5,5'-Hexachlorobiphenyl[*,1]	PCB 169	1336-36-3		0.00000003			x
2,2',3,4,4',5,5'-Heptachlorobiphenyl[*,1]	PCB 180	35065-29-3		0.03			x
2,3,3',4,4',5,5'-Heptachlorobiphenyl[*,1]	PCB 189	39635-31-9		0.00002			x
Short-chained chlorinated paraffins[*,1]	SCCPs	-	2.2	6.2			x
Medium-chained chlorinated paraffins	MCCPs	-	3.0	8.8			x
Pentachlorophenol[*,1]	PCP	87-86-5	0.5				x
Pentachloroanisole		1825-21-4	0.5				x
Hexachlorobutadiene[*,1,3]	HCBD	87-68-3	1.0				x

(1) pilot study (2013); (2) follow-up study (2014-2016); (3) WHO/UNEP breast milk monitoring programme
* listed in Stockholm Convention
[1] listed in Annex A of the Stockholm Convention (Elimination)
[2] listed in Annex B of the Stockholm Convention (Restriction)
[3] listed in Annex C of the Stockholm Convention (Unintentional production)
[4] recommended for listing in the Stockholm Convention
[5] sum of alpha-chlordane (CAS# 5103-71-9), gamma-chlordane (CAS# 5566-34-7) and oxy-chlordane (CAS# 27304-13-8); calculated as chlordane
[6] sum of o,p'-DDT (CAS# 789-02-6), p,p'-DDT (CAS# 50-29-3), p,p'-DDE (CAS# 68679-99-2) and p,p'-DDD (CAS# 72-45-8); calculated as DDT
[7] sum of endrin (CAS# 72-20-8) and endrin ketone (CAS# 53494-70-5); calculated as endrin
[8] sum of heptachlor (CAS# 76-44-8) and heptachlor epoxide (cis/trans) (CAS# 1024-57-3); calculated as heptachlor
[9] sum of alpha-endosulfan (CAS# 959-98-8), beta-endosulfan (CAS# 33213-65-9) and endosulfan sulfate (CAS# 1031-07-8)
[10] sum of parlar 26 (CAS# 142534-71-2), parlar 50 (CAS# 66860-80-8) and parlar 62 (CAS# 154159-06-5)
[11] given in ng/g milk; comprising the method detection limit (MDL)

Table S2: Correlations (Spearman) between levels of PBDE congeners in breast milk (n=18) of the pilot study (2013).

	**triBDE**	**tetraBDEs**				**pentaBDEs**				**hexaBDEs**			**heptaBDE**	**octaBDEs**			**nonaBDE**
	BDE-28	BDE-47	BDE-49	BDE-66	BDE-77	BDE-99	BDE-100	BDE-118	BDE-126	BDE-139	BDE153	BDE-154	BDE-183	BDE-196	BDE-197	BDE-203	BDE-207
**BDE-47**	0.705**																
**BDE-49**	0.656**	0.421															
**BDE-66**	0.654**	0.428	0.997**														
**BDE-77**	0.684**	0.443	0.955**	0.952**													
**BDE-99**	0.767**	0.934**	0.501*	0.504*	0.515*												
**BDE-100**	0.882**	0.866**	0.539*	0.544*	0.573*	0.920**											
**BDE-118**	0.681*	0.450	0.952**	0.955**	0.997**	0.518*	0.578*										
**BDE-126**	0.213	-0.024	0.346	0.292	0.362	0.072	0.070	0.306									
**BDE-139**	0.644**	0.442	0.828**	0.828**	0.867**	0.488*	0.545*	0.867**	0.385								
**BDE- 153**	0.609**	0.243	0.538*	0.528*	0.431	0.304	0.507*	0.421	0.047	0.370							
**BDE-154**	0.688**	0.449	0.944**	0.940**	0.989**	0.519*	0.570*	0.985**	0.306	0.800**	0.450						
**BDE-183**	0.108	0.096	0.029	0.034	-0.037	0.033	0.153	-0.032	-0.352	0.038	0.507*	-0.005					
**BDE-196**	0.069	-0.013	-0.343	-0.343	-0.420	-0.004	0.112	-0.420	-0.148	-0.323	0.411	-0.420	0.487*				
**BDE-197**	0.262	0.230	0.029	0.046	-0.086	0.134	0.305	-0.069	-0.398	0.058	0.601**	-0.092	0.570*	0.555*			
**BDE-203**	0.189	0.294	-0.322	-0.322	-0.278	0.322	0.369	-0.278	-0.148	-0.323	0.115	-0.268	-0.039	0.564*	0.372		
**BDE-207**	0.166	0.290	-0.358	-0.358	-0.382	0.225	0.216	-0.382	-0.225	-0.221	0.138	-0.382	0.122	0.520*	0.558*	0.669**	
**BDE-209**	0.138	0.219	-0.369	-0.369	-0.458	0.153	0.198	-0.458	-0.245	-0.285	0.286	-0.458	0.272	0.674**	0.682**	0.599**	0.921**

* Correlation is significant at the 0.05 level (2-tailed). ** Correlation is significant at the 0.01 level (2-tailed).

Table S3: Results on PBDE congeners and total PBDEs concentration (ranges, means, medians in ng/g lipid; detection rates in %) from selected studies in European populations.

Country	Sampling period	n	BDE-28	BDE-47	BDE-66	BDE-85	BDE-99	BDE-100	BDE-153	BDE-154	BDE-209	Total PBDEs	Reference
							**range (mean; median) [ng/g lipid]** ***detection rate***						
Sweden (Uppsala)	1996-2006	276		<0.40-16 (1.9; 1.5)			<0.12-5.2 (0.45; 0.32)	<0.10-5.1 (0.36; 0.29)	0.20-4.6 (0.64; 0.57)			0.91-28 (3.5; 2.9)[1]	[1]
Finland (Turku), Denmark (Copenhagen)	1997-2001	68	(-; 0.1)	(-; 1.12)	(-; 0.03)		(-; 0.42)	(-; 0.27)	(-; 0.81)	(-; 0.04)		(-; 104.2)[2]	[2]
Italy (Rome)	2000-2001	1[3]	0.082	1.9	0.019	0.074	0.97	0.48	0.47	0.07		4.1[4]	[3]
Norway (Tromsø)	2000-2002	10	n.d.-0.4 (0.12; 0.10) *90%*	0.42-6.12 (1.74; 1.26) *100%*			0.16-1.42 (0.49; 0.41) *100%*	0.15-0.79 (0.38; 0.40) *100%*	0.43-1.85 (0.77; 0.68) *100%*	n.d.-0.25 (0.07; 0.06) *60%*	0.05-0.72 (0.22; 0.13) *100%*	1.68-9.67 (3.80; 3.19)[5]	[4]
Czech Republic (Olomouc region)	2003	103	<0.02-0.07 (0.06; 0.07)	0.16-2.02 (0.65; 0.58)	<0.02-0.11 (0.08; 0.08)		<0.02-0.70 (0.24; 0.22)	<0.02-0.35 (0.13; 0.12)	<0.03-0.54 (0.17; 0.15)	<0.02-0.12 (0.08; 0.07)			[5]
Spain (Madrid, Vallecas District)	2003-2004	22	<0.01-0.1 (-; 0.01)	0.03-3.6 (-; 0.37)	<0.01	<0.01-3.1 (-; 0.17)	0.30-3.3 (-; 0.51)	0.18-1.9 (-; 0.58)	<0.03-3.2 (-; 0.13)	<0.005-2.0 (-; 0.02)	<0.16-52 (-; 2.9)		[6]

**Country**	**Sampling period**	**n**	**BDE-28**	**BDE-47**	**BDE-66**	**BDE-85**	**BDE-99**	**BDE-100**	**BDE-153**	**BDE-154**	**BDE-209**	**Total PBDEs**	**Reference**
						**range (mean; median) [ng/g lipid]** ***detection rate***							
Spain (Madrid, Getafe District)	2003-2004	30	<0.01	<0.003-1.2 (-; 0.22)	<0.01	<0.01-0.43 (-; 0.12)	0.15-0.72 (-; 0.38)	0.15-1.0 (-; 0.46)	<0.02-1.2 (-; 0.1)	<0.01	<0.16-33 (-; 2.8)		[6]
Norway	2003+	393 (46)[6]	<LOQ-6.8 (0.18; 0.093) *95.4%*	0.15-56 (1.7; 0.99) *100%*		<LOQ-0.89 (0.077; 0.040) *40.7%*	0.02-9.5 (0.49; 0.27) *100%*	<LOQ-6.4 (0.40; 0.25) *99.7%*	<LOQ-5.0 (0.56; 0.45) *99.5%*	<LOQ-1.2 (0.062; 0.036) *63.4%*	<LOQ-5.8 (0.61; 0.32) *76%*	0.48-82 (3.4; 2.1)[7]	[7]
Spain (Madrid)	2004	11										0.04-1.38 (0.33; -)[8]	[8]
Poland (Wielkopolska)	2004	22	<LOQ-0.33 (0.07; <LOQ)	0.31-5.62 (1.07; 1.03)			<LOQ-1.43 (0.47; 0.33)	<LOQ-0.55 (0.15; 0.05)	0.015-1.12 (0.53; 0.45)			0.08-8.4 (2.5; 2.0)[9]	[9]
France (Toulouse)	2004-2006	62-77	0.037-1.62 (0.179; 0.089)	0.343-12.0 (0.162; 1.15)		0.009-0.216 (0.046; 0.028)	0.133-5.3 (1.10; 0.527)	0.046-3.91 (0.412; 0.226)	0.288-10.5 (0.019; 0.781)	0.009-0.691 (0.097; 0.04)	0.39-6.80 (1.88; 1.62)		[10]
Spain (Madrid)	2005	9	<LOQ-0.097	0.15-0.97 (0.53; 0.54)	<LOQ-0.017	<LOQ-0.26 (0.14; 0.15)	0.32-0.69 (0.52; 0.51)	0.19-0.81 (0.55; 0.58)	0.040-0.63 (0.24; 0.16)	<LOQ-0.26 (0.061; 0.017)	0.20-5.7 (2.5; 2.7)	2.1-11 (5.5; 5.3)[10]	[11]

Country	Sampling period	n	BDE-28	BDE-47	BDE-66	BDE-85	BDE-99	BDE-100	BDE-153	BDE-154	BDE-209	Total PBDEs	Reference
			range (mean; median) [ng/g lipid]<br>*detection rate*										
			(0.032; 0.01)		(<LOQ; <LOQ)								
Germany (Munich)	2005	42	0.19-2.24 (0.66; 0.51)<br>*95% (>LOQ)*				0.03-1.34 (0.28; 0.18)<br>*83% (>LOQ)*	0.03-0.70 (0.18; 0.15)<br>*100% (>LOQ)*	0.19-2.02 (0.73; 0.61)<br>*95% (>LOQ)*			0.65-5.76 (2.03; 1.64)[11]	[12]
Slovakia[12]	2006-2007	14	(0.022; 0.017)	(0.20; 0.12)			(0.051; 0.032)	(0.056; 0.043)	(0.14; 0.12)	(0.009; 0.008)		(0.52; 0.40)[13]	[13]
Belgium (Flanders)	2009-2010	84	(-; <LOQ)	(-; 0.16)			(-; 0.06)	(-; 0.06)	(-; 0.29)	(-; 0.07)	(-; 0.65)		[14]
United Kingdom (Birmingham)	2010	35		0.17-14.65 (3.3; 2.8)<br>*100%*		<0.05-0.83 (0.08; <0.05)<br>*46%*	<0.06-3.43 (0.71; 0.69)<br>*94%*	<0.05-1.86 (0.45; 0.38)<br>*89%*	<0.06-4.57 (1.1; 0.91)<br>*97%*	<0.06-11.1 (0.30; 0.21)<br>*77%*	<0.06-0.92 (0.31; 0.25)<br>*69%*		[15]
United Kingdom	2011-2012	6	0.02-0.31 (-; 0.09)<br>*100%*	0.32-13.1 (-; 1.92)<br>*100%*	<0.03-0.13 (-; 0.03)<br>*67%*	<0.01-0.35 (-; 0.04)<br>*83%*	0.12-3.7 (-; 0.88)<br>*100%*	0.07-2.19 (-; 0.64)<br>*100%*	0.70-1.68 (-; 1.01)<br>*100%*	0.01-0.18 (-; 0.07)<br>*100%*	<0.20-1.04 (-; 0.54)<br>*83%*	1.28-22.02 (-; 5.67)[14]	[16]

Country	Sampling period	n	BDE-28	BDE-47	BDE-66	BDE-85	BDE-99	BDE-100	BDE-153	BDE-154	BDE-209	Total PBDEs	Reference
						**range (mean; median) [ng/g lipid]** ***detection rate***							
Germany	2016	100	<LOQ-0.12 (0.031; 0.029) *96%*	x-2.4 (0.31;0.20) *100%*	<LOQ-0.022 (;) *39%*	<LOQ-0.028 (;) *19%*	<LOQ-0.42 (0.086;0.063) *99%*	<LOQ-0.45 (0.076;0.054) *98%*	x-1.98 (0.46;0.38) 100%	<LOQ-0.029 (0.009;0.007) *83%*	<LOQ-104 (4.22;0.42) *96%*	x-113 (5.73;1.74)[16]	[17]
Austria (Vienna)	2013	18	n.d.-0.38 (0.11; <LOQ) *72.2%*	n.d.-6.0 (1.04; <LOQ) *66.7%*	n.d.-0.48 (0.051; n.d.) *38.9%*	n.d. *0%*	n.d.-2.4 (0.52;<LOQ) *66.7%*	n.d.-1.1 (0.22; 0.16) *77.8%*	n.d.-0.86 (0.28; 0.23) *88.9%*	n.d.-1.3 (0.12; n.d.) *33.3%*	n.d.-43 (11.8; <LOQ) *55.6%*	0.055-52 (15; 11)[15]	this study

Abbreviations: LOQ: limit of quantification; n.d.: not detected.

[1] Σ BDE-47, -99, -100, -153 and -154. [2] Σ BDE-28, -47, -66, -71, -75, -77, -85, -99, -100, -119, -138, -153, -154 and -183. [3] Pooled sample consisting of 10 donors. [4] Σ BDE-17, -28, -47, -66, -85, -99, -100, -138, -153, -154 and -183. [5] Σ BDE-28, -47, -99, -100, -153, -154 and -209. [6] BDE-209 was investigated in a subsample (n=46). [7] Σ BDE-28, -47, -99, -100, -153, -154 and -183. [8] Σ BDE-17, -28, -47, -66, -85, -99, -100, -153 and -154. [9] Σ BDE-28, -47, -99, -100, -153 and -183. [10] Σ BDE-17, -28, -47, -66, -85, -99, -100, -153, -154, -183, -184, -191, -196, -197 and -209. [11] Σ BDE-47, -99, -100, -153 and -183. [12] Breast milk samples of women from four Slovakian areas were investigated in this study. Only the results from the area with the highest sample size are included in the table. [13] Σ BDE-28, -47, -99, -100, -153, -154 and -183. [14] Σ BDE-28, -47, -49, -66, -85, -99, -100, -138, -153, -154, -183 and -209. [15] Σ BDE-28, -47, -49, -66, -77, -85, -99, -100, -118, -126, -139, -153, -154, -181, -183, -196, -197, -203, -207 and -209. [16] ∑BDE-17,-18, -47, -66, -85, -99, -100, -153, -154, -183, -196, -197, -203, -206, -207, -208, -209.

Table S4: Results on PFOA and PFOS concentrations (ranges, means, medians; detection rates in %) from selected studies in European populations.

Country (City)	Sampling period	n	PFOS [µg/l]	PFOA [µg/l]	Reference
			range (mean; median) *detection rate %*		
Hungary (Gyor)	1996-1997	13	0.096-0.639 (0.317; 0.330)		[18]
Germany (Leipzig)	1996-2006	38	0.033-0.309 (0.126; 0.123)		[18]
Sweden (Uppsala)	2004	12	0.060-0.470 (0.201; 0.166)<br>*100%*	<0.209-0.492 (0.017; -)<br>92%	[19]
Germany (Munich)	2006	19	0.028-0.239 (0.116; 0.113)		[18]
Belgium	2006	22	<0.40-28.2 (-; 2.9)	<0.30-3.5 (-; 0.3)	[20]
France	2007	48	<0.050-0.33 (0.092; 0.079)<br>*90%*	<0.070-0.224 (0.082; 0.075)<br>*98%*	[21]
Spain (Catalonia)	2007-2008	10	0.07-0.22 (0.12; 0.11)<br>*100%*	<LOD<br>*0%*	[22]
Germany (Bavaria)	2007-2008	302	<0.02-0.26 (0.06; 0.05)<br>*100%*	<0.08-0.29 (0.08; -)<br>*2%*	[23]
Spain (Barcelona)	before 2010	20	<0.012-0.865 (0.116; 0.084)<br>*95% (>LOQ)*	<0.015-0.907 (0.15; -)<br>*45%*	[24]
Belgium (Flanders)	2009-2010	40	(0.13; 0.10)<br>*100% (>LOQ)*	(0.08; 0.07)<br>*100% (>LOQ)*	[14]
Italy (Bologna)	2010	21[a]	0.015-0.288 (0.057; -)[a]<br>*90% (>LOQ)*[a]	0.024-0.241 (0.076; -)[a]<br>*81% (>LOQ)*[a]	[25]
Italy (Bologna)	2010	16[b]	0.015-0.116 (0.036; -)[b]<br>*63% (>LOQ)*[b]	0.024-0.100 (0.043; -)[b]<br>*69% (>LOQ)*[b]	[25]
France (Toulouse)	2010-2013	61	<LOD-0.376 (0.04; <0.04)<br>*82%*	<LOD-0.308 (0.041; <0.05)<br>*77%*	[26]
Italy (Siena)	before 2013	49	1.02-4.28 (0.85; -)	n.d.- 7.78 (0.16; -)	[27]

Country (City)	Sampling period	n	PFOS [µg/l]	PFOA [µg/l]	Reference
			**range (mean; median)** ***detection rate %***		
			41%	*2%*	
Netherlands	2014	50	0.045	<0.080	[28]
Czech Republic	2014	164	<0.002-0.095 (0.018; -)	<0.006-0.159 (0.034; -)	[29]
Sweden	2016	10	0.023-0.058 (0.039; -) *25%*	<0.002-0.081 (0.042; -) *25%*	[30]
Ireland	probably 2016	92	<0.02-0.12 (0.038; 0.02) *62%*	0.016-0.35 (0.13; 0.10) *100%*	[31]
Czech Republic	2017	232	<0.002-0.083 (0.014; -)	<0.003-0.16 (0.024; -)	[29]
Germany (Schleswig-Holstein)	2015-2017	80	(0,018; <0,025)	(0,016; <0,025)	[17]
Germany (Bavaria)	2016-2018	100	(0,017; <0,025)	(0,027; <0,025)	[17]
Austria (Vienna)	2013	21	0.058 – 0.31 (0.124; 0.11) *100%*	n.d.-0.83 (0.016; -) *52%*	present study
Austria (Vienna)	2014-2016	40	<LOD-0.55 (0.015; 0.012) *97.5%*	<LOQ-0.91 (0.030; 0.025) *100%*	present study

Abbreviations: LOQ: limit of quantification; LOD: limit of quantification; “-“: not available
[a] primipara
[b] multipara

Table S5: POPs detected in the Austrian pool sample within WHO/UNEP

POP concentrations in WHO/UNEP pooled breast milk sample [ng/g lipid] (n=1)		Health based values [ng/g lipid]	Daily intake via breast milk consumption [µg/kg bw/d][1]		Health based guidance values[2] (TDI, PTDI, MRL[3]) [µg/kg bw/d]
**Substance**	Concentration		Average intake	High intake	
**Aldrin**	n.d.	BE: 2,3002	-	-	TDI: 0.1[21]
**Chlordane group** (sum of α-chlordane, γ-chlordane and oxy-chlordane; calculated as chlordane)	1.5		0.0081	0.0121	PTDI: 0.5[4]<br>MRL: 0.0000006[5]
**Dieldrin**	2.0	x	0.0108	0.0161	PTDI, TDI: 0.1[6,21]<br>MRL: 0.00000005[5]
**DDT group** (sum of o,p'-DDT, p,p'-DDT, p,p'-DDE and p,p'-DDD; calculated as DDT)	120	2,300[18]	0.6452	0.9679	PTDI: 0.00001[7]<br>MRL: 0.0000005[8]<br>TDI: 10[21]
**Endrin group** (sum of endrin and endrin ketone; calculated as endrin)	<0.5		-	-	
**Heptachlor group** (sum of heptachlor and heptachlor-epoxide (cis/trans); calculated as heptachlor)	1.6		0.0086	0.0129	TDI: 0.0000001[9]<br>MRL: 0.0000001[10]
**HCB**	14.9		0.0807	0.1210	TDI: 0.17[11]<br>MRL: 0.00000007[5]
**HCH-group**					
α-HCH	<0.5		-	-	
β-HCH	23.4		0.1258	0.1887	MRL: 0.0000006[12]
γ-HCH	<0.5		-	-	
**Endosulfan group** (sum of alpha-endosulfan, beta-endosulfan, endosulfan sulfat)	<0.5		-	-	
**Toxaphene group** (sum of parlar 26, parlar 50 and parlar 62)	<0.5		-	-	
**Mirex**	<0.5		-	-	
**Hexabrombiphenyl**	<0.5		-	-	
**Pentachlorobenzene**	<0.5		-	-	TDI: 1[21]
**Pentachlorophenol**	<0.5				TDI: 6[21]
**Pentachloroanisole**	<1.0				
**Hexachlorobutadiene**	<1.0				
**p,p-Dicofol**	NA				
**Chlordecone**	<0.5		-	-	
**HBCD group**	6.0				
α-HBCD	6.0	BE: 190,000[13]	0.0323	0.0484	-

**POP concentrations in WHO/UNEP pooled breast milk sample [ng/g lipid] (n=1)**		**Health based values [ng/g lipid]**	**Daily intake via breast milk consumption [µg/kg bw/d][1]**		**Health based guidance values[2] (TDI, PTDI, MRL[3]) [µg/kg bw/d]**
**Substance**	Concentration		Average intake	High intake	
		HBM-I: 300[14,15]			
β-HBCD	<0.1		-	-	
γ-HBCD	<0.1		-	-	
**PBDEs (sum)[16]**	1.32				
BDE-17	0.0017				
BDE-28	0.0256				
BDE-47	0.412				
BDE-66	0.0067				
BDE-99	0.128				
BDE-100	0.126				
BDE-138	0.0042				
BDE-153	0.548				
BDE-154	0.0124				
BDE-183	0.0542				
**Dioxins and Furans (WHO2005-PCDD/F-TEQ)**	0.0032	BE (Dioxin-TEQ): 15[13]			TWI: 2 pg TEQ/kg bw/week[17]
2,3,7,8-TCDF	0.00028				
1,2,3,7,8-PeCDF	0.00017				
2,3,4,7,8-PeCDF	0.00354				
1,2,3,4,7,8-HxCDF	0.00103				
1,2,3,6,7,8-HxCDF	0.00092				
2,3,4,6,7,8-HxCDF	0.00050				
1,2,3,7,8,9-HxCDF	0.000039				
1,2,3,4,6,7,8-HpCDF	0.00073				
1,2,3,4,7,8,9-HpCDF	0.000037				
OCDF	0.00010				
2,3,7,8-TCDD	0.00044				
1,2,3,7,8-PeCDD	0.00103				
1,2,3,4,7,8-HxCDD	0.00054				
1,2,3,6,7,8-HxCDD	0.00218				
1,2,3,7,8,9-HxCDD	0.00065				
1,2,3,4,6,7,8-HpCDD	0.0032				
OCDD	0.0186				
**Polychlorinated biphenyls (WHO-PCB-TEQ)**	0.00231				TWI: 2 pg TEQ/kg bw/week[20]
PCB 28	0.91				
PCB 52	0.11				
PCB 101	0.18				
PCB 138	9.6				
PCB 153	17.7				
PCB 180	9.6				
sum of indicator PCB	38.0				
PCB 105	0.60				
PCB 114	0.12				
PCB 118	2.88				
PCB 123	0.025				
PCB 156	1.50				
PCB 157	0.24				
PCB 167	0.50				
PCB 189	0.14				

POP concentrations in WHO/UNEP pooled breast milk sample [ng/g lipid] (n=1)		Health based values [ng/g lipid]	Daily intake via breast milk consumption [µg/kg bw/d][1]		Health based guidance values[2] (TDI, PTDI, MRL[3]) [µg/kg bw/d]
**Substance**	Concentration		Average intake	High intake	
PCB 77	0.004				
PCB 81	0.001				
PCB 126	0.018				
PCB 169	0.010				
**WHO-mono-ortho PCB-TEQ**	0.00018				
**WHO-non-ortho PCB-TEQ**	0.00213				
**WHO-PCDD/F-PCB-TEQ**	0.00551				
**Chlorinated paraffins**	20				
SCCPs	20				TWI: 100 µg/kg bw/day[19]
MCCPs	n.n.				

[1] Daily intakes were calculated based on results substance concentrations in the Austrian WHO pooled breast milk sample according to calculation procedure published in [32] considering the following assumptions: 800 ml breast milk consumption per day for average intake, 1,200 ml breast milk consumption per day for high intake, 6.1 kg infant bodyweight. Analytical data is expressed in ng/g lipid. The measured lipid content of the pooled breast milk sample is 4.1%, which was used for the calculation. [2] Values are only given for substances found the investigated pooled breast milk sample. [3] [33]. [4] Derived based on liver toxicity in rats; source: [34]. [5] Chronic, oral; endpoint: hepatic. [6] [35]. [7] Derived based on developmental effects in rats; source: [36]. [8] MRL for p,p‘-DDT; oral, intermediate; endpoint: hepatic. [9] Derived based on histopathological changes in liver of dogs; source: [37]. [10] Oral, intermediate; endpoint: immunotoxicity. [11] [36]. [12] Oral, intermediate; endpoint: hepatic. [13] [38]. [14] [39]. [15] HBM-I value for total HBCD. [16] sum of BDE-15, -17, -28, -47, -49, -66, -75, -77, -85, -99, -100, -119, -126, -138, -153, -154, -183, -190, -196, -197, -203, -206, -207, -208 and -209. [17] [40]. [18] [41]. [19] [42]. [20] [40]. [21] [43].
Abbreviations: BE: Biomonitoring Equivalent; bw: body weight; d: day; HBM: Human Biomonitoring; MRL: minimal risk level; n.d.: not detected; POP: persistent organic pollutants; PTDI: provisional tolerable daily intake; TDI: tolerable daily intake; TEQ: toxic equivalent; TWI: tolerable weekly intake.

References

1. Lignell, S.; Aune, M.; Darnerud, P.O.; Cnattingius, S.; Glynn, A. Persistent organochlorine and organobromine compounds in mother's milk from Sweden 1996-2006: compound-specific temporal trends. *Environ. Res.* **2009**, *109*, 760–767, doi:10.1016/j.envres.2009.04.011.
2. Main, K.M.; Kiviranta, H.; Virtanen, H.E.; Sundqvist, E.; Tuomisto, J.T.; Tuomisto, J.; Vartiainen, T.; Skakkebaek, N.E.; Toppari, J. Flame retardants in placenta and breast milk and cryptorchidism in newborn boys. *Environ. Health Perspect.* **2007**, *115*, 1519–1526, doi:10.1289/ehp.9924.
3. Ingelido, A.M.; Ballard, T.; Dellatte, E.; Di Domenico, A.; Ferri, F.; Fulgenzi, A.R.; Herrmann, T.; Iacovella, N.; Miniero, R.; Päpke, O.; et al. Polychlorinated biphenyls (PCBs) and polybrominated diphenyl ethers (PBDEs) in milk from Italian women living in Rome and Venice. *Chemosphere* **2007**, *67*, 301-306, doi:10.1016/j.chemosphere.2006.05.111.
4. Polder, A.; Thomsen, C.; Lindström, G.; Løken, K.B.; Skaare, J.U. Levels and temporal trends of chlorinated pesticides, polychlorinated biphenyls and brominated flame retardants in individual human breast milk samples from Northern and Southern Norway. *Chemosphere* **2008**, *73*, 14–23, doi:10.1016/j.chemosphere.2008.06.002.
5. Kazda, R.; Hajšlová, J.; Poustka, J.; Čajka, T. Determination of polybrominated diphenyl ethers in human milk samples in the Czech Republic. *Anal. Chim. Acta* **2004**, *520*, 237–243, doi:10.1016/j.aca.2004.04.069.
6. Gómara, B.; Herrero, L.; Ramos, J.J.; Mateo, J.R.; Fernández, M.A.; García, J.F.; González, M.J. Distribution of polybrominated diphenyl ethers in human umbilical cord serum, paternal serum, maternal serum, placentas, and breast milk from Madrid population, Spain. *Environ. Sci. Technol.* **2007**, *41*, 6961–6968, doi:10.1021/es0714484.
7. Thomsen, C.; Stigum, H.; Frøshaug, M.; Broadwell, S.L.; Becher, G.; Eggesbø, M. Determinants of brominated flame retardants in breast milk from a large scale Norwegian study. *Environ. Int.* **2010**, *36*, 68–74, doi:10.1016/j.envint.2009.10.002.
8. Bordajandi, L.R.; Abad, E.; González, M.J. Occurrence of PCBs, PCDD/Fs, PBDEs and DDTs in Spanish breast milk: enantiomeric fraction of chiral PCBs. *Chemosphere* **2008**, *70*, 567–575, doi:10.1016/j.chemosphere.2007.07.019.
9. Jaraczewska, K.; Lulek, J.; Covaci, A.; Voorspoels, S.; Kaluba-Skotarczak, A.; Drews, K.; Schepens, P. Distribution of polychlorinated biphenyls, organochlorine pesticides and

polybrominated diphenyl ethers in human umbilical cord serum, maternal serum and milk from Wielkopolska region, Poland. *Sci. Total Environ.* **2006**, *372*, 20–31, doi:10.1016/j.scitotenv.2006.03.030.

10. Antignac, J.-P.; Cariou, R.; Zalko, D.; Berrebi, A.; Cravedi, J.-P.; Maume, D.; Marchand, P.; Monteau, F.; Riu, A.; Andre, F.; et al. Exposure assessment of French women and their newborn to brominated flame retardants: determination of tri- to deca- polybromodiphenylethers (PBDE) in maternal adipose tissue, serum, breast milk and cord serum. *Environ. Pollut.* **2009**, *157*, 164–173, doi:10.1016/j.envpol.2008.07.008.
11. Gómara, B.; Herrero, L.; Pacepavicius, G.; Ohta, S.; Alaee, M.; González, M.J. Occurrence of co-planar polybrominated/chlorinated biphenyls (PXBs), polybrominated diphenyl ethers (PBDEs) and polychlorinated biphenyls (PCBs) in breast milk of women from Spain. *Chemosphere* **2011**, *83*, 799–805, doi:10.1016/j.chemosphere.2011.02.080.
12. Raab, U.; Preiss, U.; Albrecht, M.; Shahin, N.; Parlar, H.; Fromme, H. Concentrations of polybrominated diphenyl ethers, organochlorine compounds and nitro musks in mother's milk from Germany (Bavaria). *Chemosphere* **2008**, *72*, 87–94, doi:10.1016/j.chemosphere.2008.01.053.
13. Chovancová, J.; Čonka, K.; Kočan, A.; Sejáková, Z.S. PCDD, PCDF, PCB and PBDE concentrations in breast milk of mothers residing in selected areas of Slovakia. *Chemosphere* **2011**, *83*, 1383–1390, doi:10.1016/j.chemosphere.2011.02.070.
14. Croes, K.; Colles, A.; Koppen, G.; Govarts, E.; Bruckers, L.; van de Mieroop, E.; Nelen, V.; Covaci, A.; Dirtu, A.C.; Thomsen, C.; et al. Persistent organic pollutants (POPs) in human milk: a biomonitoring study in rural areas of Flanders (Belgium). *Chemosphere* **2012**, *89*, 988–994, doi:10.1016/j.chemosphere.2012.06.058.
15. Abdallah, M.A.-E.; Harrad, S. Polybrominated diphenyl ethers in UK human milk: implications for infant exposure and relationship to external exposure. *Environ. Int.* **2014**, *63*, 130–136, doi:10.1016/j.envint.2013.11.009.
16. Bramwell, L.; Fernandes, A.; Rose, M.; Harrad, S.; Pless-Mulloli, T. PBDEs and PBBs in human serum and breast milk from cohabiting UK couples. *Chemosphere* **2014**, *116*, 67–74, doi:10.1016/j.chemosphere.2014.03.060.
17. Fromme, H.; Fuchs, V.; Albrecht, M.; Aschenbrenner, B.; Röhl, C.; Janitzki, N.; Herber-Jonat, S.; Wöckner, M.; Völkel, W.; Flemmer, A.W.; et al. Polychlorinated dioxins and dibenzofurans

(PCDD/F), polybrominated dioxins and dibenzofurans (PBDD/F), polychlorinated biphenyls (PCB), polybrominated diphenyl ethers (PBDE), and per- and polyfluoroalkyl substances (PFAS) in German breast milk samples (LUPE 8). *Sci. Total Environ.* **2022**, *825*, 154066, doi:10.1016/j.scitotenv.2022.154066.

18. Völkel, W.; Genzel-Boroviczény, O.; Demmelmair, H.; Gebauer, C.; Koletzko, B.; Twardella, D.; Raab, U.; Fromme, H. Perfluorooctane sulphonate (PFOS) and perfluorooctanoic acid (PFOA) in human breast milk: results of a pilot study. *International Journal of Hygiene and Environmental Health* **2008**, *211*, 440–446, doi:10.1016/j.ijheh.2007.07.024.
19. Kärrman, A.; Ericson, I.; van Bavel, B.; Darnerud, P.O.; Aune, M.; Glynn, A.; Lignell, S.; Lindström, G. Exposure of perfluorinated chemicals through lactation: levels of matched human milk and serum and a temporal trend, 1996-2004, in Sweden. *Environ. Health Perspect.* **2007**, *115*, 226–230, doi:10.1289/ehp.9491.
20. Roosens, L.; D'Hollander, W.; Bervoets, L.; Reynders, H.; van Campenhout, K.; Cornelis, C.; van den Heuvel, R.; Koppen, G.; Covaci, A. Brominated flame retardants and perfluorinated chemicals, two groups of persistent contaminants in Belgian human blood and milk. *Environ. Pollut.* **2010**, *158*, 2546–2552, doi:10.1016/j.envpol.2010.05.022.
21. Antignac, J.-P.; Veyrand, B.; Kadar, H.; Marchand, P.; Oleko, A.; Le Bizec, B.; Vandentorren, S. Occurrence of perfluorinated alkylated substances in breast milk of French women and relation with socio-demographical and clinical parameters: results of the ELFE pilot study. *Chemosphere* **2013**, *91*, 802–808, doi:10.1016/j.chemosphere.2013.01.088.
22. Kärrman, A.; Domingo, J.L.; Llebaria, X.; Nadal, M.; Bigas, E.; van Bavel, B.; Lindström, G. Biomonitoring perfluorinated compounds in Catalonia, Spain: concentrations and trends in human liver and milk samples. *Environ. Sci. Pollut. Res. Int.* **2010**, *17*, 750–758, doi:10.1007/s11356-009-0178-5.
23. Raab, U.; Albrecht, M.; Preiss, U.; Völkel, W.; Schwegler, U.; Fromme, H. Organochlorine compounds, nitro musks and perfluorinated substances in breast milk - results from Bavarian Monitoring of Breast Milk 2007/8. *Chemosphere* **2013**, *93*, 461–467, doi:10.1016/j.chemosphere.2013.06.013.
24. Llorca, M.; Farré, M.; Picó, Y.; Teijón, M.L.; Alvarez, J.G.; Barceló, D. Infant exposure of perfluorinated compounds: levels in breast milk and commercial baby food. *Environ. Int.* **2010**, *36*, 584–592, doi:10.1016/j.envint.2010.04.016.

25. Barbarossa, A.; Masetti, R.; Gazzotti, T.; Zama, D.; Astolfi, A.; Veyrand, B.; Pession, A.; Pagliuca, G. Perfluoroalkyl substances in human milk: a first survey in Italy. *Environ. Int.* **2013**, *51*, 27–30, doi:10.1016/j.envint.2012.10.001.
26. Cariou, R.; Veyrand, B.; Yamada, A.; Berrebi, A.; Zalko, D.; Durand, S.; Pollono, C.; Marchand, P.; Leblanc, J.-C.; Antignac, J.-P.; et al. Perfluoroalkyl acid (PFAA) levels and profiles in breast milk, maternal and cord serum of French women and their newborns. *Environ. Int.* **2015**, *84*, 71–81, doi:10.1016/j.envint.2015.07.014.
27. Guerranti, C.; Perra, G.; Corsolini, S.; Focardi, S.E. Pilot study on levels of perfluorooctane sulfonic acid (PFOS) and perfluorooctanoic acid (PFOA) in selected foodstuffs and human milk from Italy. *Food Chemistry* **2013**, *140*, 197–203, doi:10.1016/j.foodchem.2012.12.066.
28. Zeilmaker, M.J.; Moermond, C.; Brandon, E.; Hoogerhuis, P.; Razenberg, L.; Janssen, M. Persistent organic pollutants in human milk in the Netherlands. *National Institute for Public Health and the Environment* **2020**.
29. Černá, M.; Grafnetterová, A.P.; Dvořáková, D.; Pulkrabová, J.; Malý, M.; Janoš, T.; Vodrážková, N.; Tupá, Z.; Puklová, V. Biomonitoring of PFOA, PFOS and PFNA in human milk from Czech Republic, time trends and estimation of infant's daily intake. *Environ. Res.* **2020**, *188*, 109763, doi:10.1016/j.envres.2020.109763.
30. Awad, R.; Zhou, Y.; Nyberg, E.; Namazkar, S.; Yongning, W.; Xiao, Q.; Sun, Y.; Zhu, Z.; Bergman, Å.; Benskin, J.P. Emerging per- and polyfluoroalkyl substances (PFAS) in human milk from Sweden and China. *Environ. Sci.: Processes Impacts* **2020**, *22*, 2023–2030, doi:10.1039/D0EM00077A.
31. Abdallah, M.A.-E.; Wemken, N.; Drage, D.S.; Tlustos, C.; Cellarius, C.; Cleere, K.; Morrison, J.J.; Daly, S.; Coggins, M.A.; Harrad, S. Concentrations of perfluoroalkyl substances in human milk from Ireland: Implications for adult and nursing infant exposure. *Chemosphere* **2020**, *246*, 125724, doi:10.1016/j.chemosphere.2019.125724.
32. EFSA. European Food Safety Authority. Scientific Opinion on Polybrominated Diphenyl Ethers (PBDEs) in Food. *EFSA J.* **2011**, *9*, doi:10.2903/j.efsa.2011.2156.
33. ATSDR. Agency for Toxic Substances and Disease Registry. Minimal Risk Levels (MRLs) List. **2018**.

34. EFSA. European Food Safety Authority. Chlordane as undesirable substance in animal feed - Scientific Opinion of the Panel on Contaminants in the Food Chain. *EFSA J.* **2007**, *5*, 582, doi:10.2903/j.efsa.2007.582.
35. EFSA. European Food Safety Authority. Opinion of the Scientific Panel on contaminants in the food chain [CONTAM] related to aldrin and dieldrin as undesirable substance in animal feed. *EFSA J.* **2005**, *3*, 285, doi:10.2903/j.efsa.2005.285.
36. EFSA. European Food Safety Authority. Opinion of the Scientific Panel on contaminants in the food chain [CONTAM] related to DDT as an undesirable substance in animal feed. *EFSA J.* **2006**, *4*, doi:10.2903/j.efsa.2006.433.
37. EFSA. European Food Safety Authority. Opinion of the Scientific Panel on contaminants in the food chain [CONTAM] related heptachlor as an undesirable substance in animal feed. *EFSA J.* **2007**, *5*, doi:10.2903/j.efsa.2007.478.
38. WHO. World Health Organization. Human biomonitoring: facts and figures.: Copenhagen: WHO Regional Office for Europe **2015**.
39. UBA. Umweltbundesamt Deutschland. FACTSHEET HBM value for HBCD **2015**.
40. EFSA. European Food Safety Authority. Risk for animal and human health related to the presence of dioxins and dioxin-like PCBs in feed and food. *EFSA J.* **2018**, *16*, e05333, doi:10.2903/j.efsa.2018.5333.
41. WHO. World Health Organization. Pesticide residues in food - 2000: Toxicological evaluations **2001**.
42. Schrenk, D.; Bignami, M.; Bodin, L.; Chipman, J.K.; Del Mazo, J.; Grasl-Kraupp, B.; Hogstrand, C.; Hoogenboom, L.R.; Leblanc, J.-C.; Nebbia, C.S.; et al. Risk assessment of chlorinated paraffins in feed and food. *EFSA J.* **2020**, *18*, e05991, doi:10.2903/j.efsa.2020.5991.
43. Health Canada. Non-Carcinogen Tolerable Daily Intake (TDI) Values from Health Canada **2007**.
